# High-Glucose Microenvironment Promotes Canine Osteosarcoma Cell Stemness via the HBP/O-GlcNAc Signaling Axis

**DOI:** 10.3390/cells15151359

**Published:** 2026-07-28

**Authors:** Weiqian Wang, Bingsong Yang, Guangmin Zhang, Meimei Wang, Junping Sun, Siyao Li, Huijie Kang, Qingdian Hou, Pujun Li, Honggang Fan, Jichen Sha

**Affiliations:** Heilongjiang Provincial Key Laboratory of Pathogenic Mechanism for Animal Disease and Comparative Medicine, College of Veterinary Medicine, Northeast Agricultural University, Harbin 150036, China; weiqianwang784@163.com (W.W.); 18945053264@163.com (B.Y.); minzai646924@163.com (G.Z.); 18804633178@163.com (M.W.); 17733266902@163.com (J.S.); dylisiyao@163.com (S.L.); hj_kang8436@163.com (H.K.); 15146613921@163.com (Q.H.); pujunli1024@163.com (P.L.)

**Keywords:** canine osteosarcoma, tumor stemness, hexosamine biosynthetic pathway (HBP), O-GlcNAcylation, high-glucose microenvironment

## Abstract

**Highlights:**

**What are the main findings?**

- Glucose gradient culture and bidirectional OGT/OGA intervention experiments demonstrated that O-GlcNAcylation positively regulates the malignant biological behaviors and stem-like properties of canine osteosarcoma cells.
- Untargeted metabolomics and O-GlcNAc-modified proteomics identified aberrant activation of the upstream hexosamine biosynthetic pathway (HBP) and revealed differentially modified proteins, including TLE3, NCOR1, and NOTCH2, which are significantly enriched in canonical stemness-associated pathways such as Wnt and Notch signaling.

**What are the implications of the main findings?**

- This study establishes the HBP/O-GlcNAc axis as a critical metabolic switch linking high-glucose signals to osteosarcoma stemness, and proposes potential therapeutic targets based on O-GlcNAc-modified proteomic profiling.
- The experimental data from canine osteosarcoma provide important translational evidence from a comparative medicine perspective, facilitating human osteosarcoma translational research and targeted drug development.

**Abstract:**

Osteosarcoma (OS) is characterized by high metastatic potential and marked chemoresistance, with cancer stem cells (CSCs) serving as major drivers of malignant progression. Canine osteosarcoma (cOS) is considered an ideal comparative medicine model for human osteosarcoma (hOS). Accumulating evidence indicates that aberrant glucose metabolism and hexosamine biosynthetic pathway (HBP, hexosamine biosynthetic pathway)/O-linked N-acetylglucosamine (O-GlcNAc)ylation are involved in tumor progression; however, the precise mechanisms by which they regulate stemness in canine osteosarcoma cells remain unclear. In this study, we comprehensively employed glucose gradient culture, untargeted metabolomics, O-GlcNAc-modified proteomics, in vitro gene silencing, and a subcutaneous xenograft model in nude mice. Cellular functional assays revealed that high glucose significantly enhanced malignant phenotypes and stemness properties of canine osteosarcoma cells. Metabolomic analyses confirmed aberrant activation of the HBP in osteosarcoma cells. Further experiments demonstrated that high glucose enhances HBP flux and O-GlcNAcylation in a dose-dependent manner; silencing of glutamine-fructose-6-phosphate transaminase 1 (GFPT1), O-GlcNAc transferase (OGT), and O-GlcNAcase (OGA) verified that both the HBP pathway and O-GlcNAcylation positively regulate malignant biological behaviors and stemness maintenance. In vivo tumorigenesis assays demonstrated that OGT knockdown markedly suppressed osteosarcoma growth. O-GlcNAc-modified proteomics identified transducin-like enhancer of split 3 (TLE3), nuclear receptor corepressor 1 (NCOR1), and neurogenic locus notch homolog protein 2 (NOTCH2) as key differentially modified proteins, predominantly enriched in the Wingless/Integrated (Wnt) and Notch signaling pathways. Collectively, our findings demonstrate that high glucose activates the HBP pathway, elevates global O-GlcNAcylation levels, and modifies TLE3/NCOR1/NOTCH2, thereby promoting stemness maintenance in canine osteosarcoma stem cells. This study provides novel metabolic targets for precision therapy of osteosarcoma.

## 1. Introduction

Osteosarcoma (OS) is a malignant bone tumor originating from mesenchymal tissue, pathologically characterized by the production of osteoid or immature bone by tumor cells [1]. Arising from malignant transformation of osteoblasts or mesenchymal stem cells, OS preferentially occurs in the metaphyses of long bones in the extremities and is associated with rapid growth, early pulmonary metastasis, strong chemoresistance, and high malignancy [1]. In human medicine, OS is the most common primary malignant bone tumor in children and adolescents. Although treatment strategies have evolved from amputation to limb-sparing surgery combined with neoadjuvant chemotherapy, the 5-year survival rate remains only approximately 20% due to uncontrolled recurrence and metastasis [2], underscoring the urgent need for novel therapeutic approaches.

In veterinary practice, canine OS similarly exhibits high incidence and aggressive behavior, with an incidence rate 10–50 times that in humans; approximately 80% of canine bone tumors are diagnosed as OS [3]. This disease predominantly affects the weight-bearing bones of the limbs in large-breed dogs, and about 85% of cases already present with pulmonary metastases at the time of diagnosis. The median survival time after amputation alone is only 4 months, and even with adjuvant chemotherapy, survival rarely exceeds 1 year [4]. The pronounced heterogeneity of OS and the existence of cancer stem cells (CSCs) are key factors contributing to poor therapeutic outcomes and dismal prognosis [4]. Therefore, elucidating the molecular mechanisms governing stemness regulation in canine OS and identifying novel therapeutic targets are of significant clinical importance. Notably, canine OS shares high similarities with human OS in terms of clinical phenotype, molecular genetic features, and tumor microenvironment, with a genetic homology of up to 99%, making it an ideal comparative medicine model for human OS research [5]. In-depth investigation of canine OS pathogenesis not only offers new therapeutic strategies for companion animals and working dogs but also provides translational evidence for precision therapy in human OS [6].

Cancer stem cells are a subpopulation of cells within tumors that possess self-renewal and unlimited proliferative capacity, and are considered central drivers of tumor initiation, metastasis, recurrence, and therapy resistance [7]. CSCs highly express stemness transcription factors such as SOX2, NANOG, and OCT4, and exhibit characteristics including high telomerase activity, strong anti-apoptotic capacity, and active DNA damage repair mechanisms [8]. Emerging evidence suggests that stemness is not a fixed phenotype but a reversible functional state regulated by the microenvironment, and this plasticity allows CSCs to flexibly switch between stem and differentiated states [9]. In canine OS, studies have confirmed the presence of CSC subpopulations in cell lines such as D-17, UWOS-1, and UWOS-2 [10]; these cells show elevated expression of stemness regulators like BMI1 and exhibit significantly enhanced clonogenic and tumorigenic capacities [11]. Moreover, canine OS stem cells display epithelial–mesenchymal transition (EMT) features, with high expression of ZEB1, Vimentin, and other proteins, forming a “stemness–EMT” vicious cycle that further promotes invasion and metastasis [12]. Additionally, CSCs are a major cause of chemoradiotherapy resistance in OS; canine OS stem cells exhibit markedly increased resistance to doxorubicin [13] and survive radiotherapy at much higher rates than non-stem tumor cells [14]. Hence, targeting CSC stemness regulation represents a pivotal direction for improving canine OS prognosis.

Metabolic reprogramming is a hallmark of cancer. Glucose metabolic remodeling, exemplified by aerobic glycolysis (the Warburg effect), not only provides energy and biosynthetic precursors for rapid tumor proliferation but also actively regulates the stemness of cancer stem cells [15,16]. Studies have shown that glucose deprivation significantly attenuates stemness, proliferation, and drug resistance in OS cells [17], while hyperglycemia is closely associated with increased tumor malignancy. Canine OS stem cells exhibit markedly enhanced glucose uptake, suggesting that glucose metabolism is a key regulator of stemness [18]. Key glycolytic enzymes, including HK2, PKM2, PFKFB3, and PFKFB4, directly modulate CSC stemness [19,20,21]; metabolic signals can also influence stemness through pathways such as Wnt/β-catenin and Hippo-YAP/TAZ [22,23]; and metabolites like lactate act as signaling molecules that reinforce stemness phenotypes [23,24]. Therefore, targeting glucose metabolism is considered a rational strategy to reverse OS stemness and overcome therapeutic resistance [25,26].

The hexosamine biosynthetic pathway (HBP) is a critical branch of glucose metabolism that integrates nutritional signals from glucose, glutamine, and acetyl-CoA to generate uridine diphosphate N-acetylglucosamine (UDP-GlcNAc) [26]. UDP-GlcNAc serves as the sole donor for protein O-linked N-acetylglucosamine (O-GlcNAc)ylation. O-GlcNAcylation, catalyzed by O-GlcNAc transferase (OGT) and removed by O-GlcNAcase (OGA), is a dynamic and reversible post-translational modification [27,28] that broadly regulates transcription, signal transduction, cell cycle progression, and metabolism [29,30,31]. In cancer, aberrant HBP activation and elevated O-GlcNAcylation promote proliferation, metastasis, EMT, and drug resistance [32]. In OS, high OGT expression facilitates cell proliferation, EMT, and chemoresistance [33]. Accumulating evidence indicates that the HBP/O-GlcNAc axis serves as a core metabolic hub for maintaining CSC stemness [34]; this pathway has been demonstrated to regulate CSC self-renewal and tumorigenic capacity in hepatocellular carcinoma, leukemia, and glioblastoma [35,36]. However, it is worth noting that the function of O-GlcNAcylation in tumors is context-dependent; some studies suggest that it may exert tumor-suppressive effects in certain normal tissues, and excessive modification under specific conditions can even induce cellular senescence or apoptosis. This implies that the pro-stemness effect of this axis may be tissue-specific, and the downstream key targets as well as the causal relationship with classical stemness pathways remain controversial [33,34]. Nevertheless, whether the high-glucose microenvironment regulates canine OS stem cell stemness through the HBP/O-GlcNAc signaling axis remains largely unknown.

Based on the above background, we propose the scientific hypothesis that a high-glucose microenvironment activates the HBP, enhances O-GlcNAcylation, and thereby promotes stemness maintenance and malignant progression of canine OS stem cells. The aims of this study are: (1) to define the regulatory effects of high glucose on the stemness phenotype of canine OS cells; (2) to screen and identify key metabolites and target proteins involved in HBP/O-GlcNAc axis-mediated stemness regulation using metabolomics and O-GlcNAc-modified proteomics; and (3) to validate the functional role of the HBP/O-GlcNAc signaling axis in canine OS stemness maintenance at both in vitro and in vivo levels. We anticipate that our findings will confirm that high glucose activates the HBP, elevates global O-GlcNAcylation, and consequently promotes stemness maintenance in canine OS cells. Meanwhile, O-GlcNAc-modified proteomics will identify differentially modified proteins that are enriched in stemness-associated pathways such as Wnt/Notch, suggesting their potential as key downstream candidate targets of the HBP/O-GlcNAc axis. This study will provide novel mechanistic insights into high glucose-driven malignant progression of canine OS from the perspective of metabolism–post-translational modification crosstalk, offer potential therapeutic targets for CSCs, and establish a theoretical foundation for human–canine comparative medicine research.

## 2. Materials and Methods

### 2.1. Cell Lines and Culture Conditions

The canine osteosarcoma (OS) cell line McKinley was kindly provided by the Flint Animal Cancer Center, Colorado State University, USA. The canine osteoblast cell line PEKCC-2382 was purchased from the Pet Cell Resource Center (PETCC). HEK-293T cells (ATCC, Manassas, VA, USA) were used for lentiviral packaging. McKinley and 293T cells were cultured in high-glucose DMEM (Kaiji, Nanjing, China, catalog No. KGM12800N) supplemented with 10% fetal bovine serum (FBS, Vivacell, Ashkelon, Israel, catalog No. C04001), 100 U/mL penicillin, and 100 μg/mL streptomycin (Meilun, Dalian, China, catalog No. MA0110). The osteoblast cell line was maintained in osteoblast complete medium (Procell, Wuhan, China, catalog No. CM-H111) containing 10% FBS and antibiotics. All cells were cultured in a humidified incubator (BB150-2TCS, Thermo Fisher Scientific, Waltham, MA, USA) at 37 °C with 5% CO_2_ and were passaged using 0.25% trypsin-EDTA (Beyotime, Shanghai, China, catalog No. C0201) upon reaching 80–90% confluence.

### 2.2. Animal Experiments

SPF-grade BALB/c-nude male mice (3 weeks old, body weight 18–20 g) were purchased from Liaoning Changsheng Biotechnology Co., Ltd. (Benxi, China). Animals were housed in SPF-grade individually ventilated cage systems under controlled conditions: temperature (22 ± 2) °C, relative humidity 50–60%, and a 12 h light/dark cycle, with free access to irradiated sterile feed and acidified water. All animal experiments were approved by the Laboratory Animal Management and Use Committee of Northeast Agricultural University (Ethics Approval No.: NEAUEC20230379, dated 1 January 2024).

For the subcutaneous tumorigenesis assay, the mice were randomly divided into two groups (sh-NC and sh-OGT), with six mice per group. Male mice were selected to exclude the potential confounding effects of cyclical estrogen fluctuations on tumor growth and bone metabolism, thereby ensuring more stable and reproducible experimental outcomes. Canine OS cells at logarithmic growth phase (1 × 10^6^ cells/100 μL PBS) were injected subcutaneously into the right axilla of nude mice. Tumor volumes were measured every 7 days and calculated using the formula V = 0.5 × length × width^2^. At the end of the experiment, mice were euthanized; tumors were excised, weighed, and fixed for subsequent histological analyses.

### 2.3. Cellular Functional Assays

Cell Viability Assay (CCK-8): Cell viability was assessed using the CCK-8 kit (Meilun, Dalian, China, catalog No. MA0218). Cells were seeded into 96-well plates at a density of 5 × 10^3^ cells/well in 100 μL of culture medium, with five replicate wells per condition per time point. Following the indicated treatments, 10 μL of CCK-8 reagent (diluted 1:10 in culture medium) was added to each well, and plates were incubated at 37 °C for 2 h. The absorbance at 450 nm was measured using a microplate reader (SpectraMax iD3, Molecular Devices, San Jose, CA, USA). Cell viability curves were generated by measuring absorbance at 0, 24, 48, and 72 h, and each experiment was independently repeated three times.

Colony Formation Assay: Cells were seeded into 6-well plates at a density of 800 cells/well in 2 mL of complete medium and cultured for 8 days, with medium changed every 3 days. Colonies were fixed with 4% paraformaldehyde (Beyotime, Shanghai, China, catalog No. P0099) for 15 min, stained with 0.1% crystal violet (Biosharp, Hefei, China, catalog No. BL802A) for 20 min at room temperature, and air-dried. Colonies containing ≥50 cells were counted independently by two observers under a light microscope (ECLIPSE E100, Nikon, Tokyo, Japan).

Wound Healing Assay: Cells were seeded into 6-well plates at a density of 5 × 10^5^ cells/well and cultured until reaching 90% confluence. A straight scratch was made using a sterile 10-μL pipette tip. After washing twice with PBS to remove detached cells, cells were cultured in medium containing 2% FBS. Images were captured at 0, 4, 8, and 12 h using an inverted fluorescence microscope (ECLIPSE TS2, Nikon, Tokyo, Japan) equipped with a digital camera. Wound closure rates were calculated using ImageJ v1.43f software (National Institutes of Health, Bethesda, MD, USA) with the MRI Wound Healing Tool plugin, and the percentage of wound closure was calculated as (1 − [wound area at indicated time/wound area at 0 h]) × 100%.

Transwell Migration and Invasion Assays: Migration and invasion assays were performed using 24-well Transwell chambers with 8.0-μm pore size polycarbonate membranes (Corning, Corning, NY, USA, catalog No. 3422). For invasion assays, the upper chambers were pre-coated with 50 μL of Matrigel (Halowa, Suzhou, China, catalog No. 8226) diluted 1:8 in serum-free medium and incubated at 37 °C for 2 h to allow gel solidification; this step was omitted for migration assays. Cells were serum-starved for 6 h, harvested, and resuspended in serum-free DMEM at a density of 2 × 10^4^ cells/200 μL. The cell suspension (200 μL) was added to the upper chamber, while the lower chamber contained 600 μL of DMEM supplemented with 10% FBS as a chemoattractant. After incubation at 37 °C for 24 h, the membranes were fixed with methanol for 15 min and stained with 0.1% crystal violet for 20 min. Non-migrated or non-invaded cells on the upper surface of the membrane were gently removed with a cotton swab. Cells on the lower surface were counted in five randomly selected high-power fields (200×) under an inverted light microscope (ECLIPSE TS2, Nikon, Japan). Each experiment was performed in triplicate.

Tumor Sphere Formation Assay: Cells were seeded at a density of 5 × 10^4^ cells/mL in ultra-low attachment 6-well plates (Corning, USA, catalog No. 3471) and cultured in DMEM/F12 stem cell medium (Kaiji, China, catalog No. KGM12500N) supplemented with 20 ng/mL recombinant human EGF (Meilun, China, catalog No. MB8218), 20 ng/mL recombinant human bFGF (Meilun, China, catalog No. MB2458), 1× B27 supplement (Meilun, China, catalog No. PWL043), and 0.4% bovine serum albumin (BSA, Beyotime, China, catalog No. B885114). After 6 days of culture, spheres with a diameter ≥75 μm were observed and counted under an inverted microscope (ECLIPSE TS2, Nikon, Japan) at 40× and 100× magnifications. Sphere-forming efficiency was calculated as the number of spheres formed per 1000 cells seeded.

Cell Cycle Analysis: Cells were harvested, washed twice with PBS, and fixed overnight in 70% ethanol at 4 °C. Fixed cells were washed with PBS and incubated with 100 μg/mL RNase A (Beyotime, China, catalog No. D7070S) at 37 °C for 30 min, followed by staining with 50 μg/mL propidium iodide (PI, Beyotime, China, catalog No. C1008S) at room temperature for 30 min in the dark. A minimum of 1 × 10^5^ cells per sample was analyzed using a flow cytometer (FACSCelesta, BD Biosciences, San Jose, CA, USA). Cell cycle distribution (G1, S, and G2/M phases) was determined using ModFit LT v5.0 software (Verity Software House, Topsham, ME, USA). Each experiment was independently repeated three times.

### 2.4. Gene Intervention

siRNA Transient Transfection: Specific siRNAs targeting canine GFPT1 and OGA genes, along with a negative control (siNC), were designed and synthesized by Genepharma (Shanghai, China). The siRNA sequences are listed in Table 1. Cells were seeded into 6-well plates at a density of 2 × 10^5^ cells/well and cultured overnight to reach 60–70% confluence. Transfection was performed using siRNA-Mate Plus transfection reagent (Genepharma, China) according to the manufacturer’s protocol, with a final siRNA concentration of 50 nM. After 48 h of transfection, knockdown efficiency was evaluated by qPCR and Western blot. Based on the highest silencing efficiency, siGFPT1-860 and siOGA-1136 (sequences shown in Table 1) were selected for subsequent functional experiments.

Lentivirus-Mediated shRNA Stable Knockdown: shRNA lentiviral vectors targeting canine OGT (LV3 H1/GFP&Puro) were constructed by Genepharma (Shanghai, China), with target sequences shown in Table 2. Lentiviral particles were produced by co-transfecting HEK-293T cells with the shRNA vector and packaging plasmids (pMD2.G and psPAX2) using PolyFast transfection reagent (MCE, Monmouth Junction, NJ, USA, catalog No. HY-K1057). Viral supernatants were collected at 48 h and 72 h post-transfection, filtered through 0.45-μm filters, and concentrated by ultracentrifugation. McKinley cells at 70% confluence were infected with lentivirus for 24 h. Stably transduced cells were selected with 2 μg/mL puromycin (MCE, USA, catalog No. HY-K1057) for one week to obtain stable knockdown cell lines. Knockdown efficiency was validated by qPCR and Western blot, and shOGT-1000 (sequence shown in Table 2) was selected for establishing stable OGT-knockdown cells.

### 2.5. Metabolomics and O-GlcNAcylated Proteomics

Untargeted Metabolomics: Untargeted metabolomic profiling and data analysis were performed by Hangzhou Kaitai Biotechnology Co., Ltd., Hangzhou, China. The workflow included metabolite extraction, ultra-high-performance liquid chromatography-tandem mass spectrometry (UHPLC-MS/MS) detection, data preprocessing, multivariate statistical analysis, differential metabolite screening, and KEGG pathway enrichment analysis. Raw data have been deposited in the MetaboLights database (accession numbers will be provided during the review process).

O-GlcNAcylated Proteomics: O-GlcNAc-modified proteomic profiling and data analysis were conducted by Shanghai Applied Protein Technology Co., Ltd., Shanghai, China. The workflow included protein extraction, tryptic digestion, enrichment of O-GlcNAcylated peptides, liquid chromatography-tandem mass spectrometry (LC-MS/MS) detection, modified site identification, quantitative analysis, and bioinformatics analysis (GO/KEGG enrichment and protein–protein interaction network analysis). Raw data have been deposited in the ProteomeXchange database (accession numbers will be provided during the review process).

### 2.6. Protein Detection

Western Blotting: Total protein was extracted from cells or tissues using RIPA lysis buffer (Beyotime, China, catalog No. P0013B) containing 1 mM PMSF (Beyotime, China, catalog No. ST505) and a phosphatase inhibitor cocktail (Beyotime, China, catalog No. P1045). Protein concentrations were determined using the BCA protein assay kit (Meilun, China, catalog No. MA0082). Equal amounts of protein (10 μg per lane) were separated by 8–12% SDS-PAGE (depending on the molecular weight of the target proteins) and wet-transferred onto PVDF membranes (Cytiva, Shanghai, China, catalog No. 10600058) at 300 mA for 90 min at 4 °C. Membranes were blocked with 5% non-fat milk in TBST (Tris-buffered saline containing 0.1% Tween-20) for 2 h at room temperature and incubated with primary antibodies overnight at 4 °C with gentle shaking. The primary antibodies and their dilution ratios were as follows: anti-GFPT1 (1:1000, Proteintech, Wuhan, China, catalog No. 14132-1-AP), anti-OGT (1:2500, Proteintech, China, catalog No. 11576-2-AP), anti-OGA (1:2500, Hao Yuan, Shanghai, China, catalog No. HY-P82296), anti-O-GlcNAc (1:5000, Proteintech, China, catalog No. 65292-1-Ig), anti-SOX2 (1:500, Hao Yuan, China, catalog No. HY-P80334), anti-NANOG (1:5000, Hao Yuan, China, catalog No. HY-P84804), anti-OCT4 (1:5000, Hao Yuan, China, catalog No. HY-P80770), anti-ALDH1 (1:5000, Proteintech, China, catalog No. 15910-1-AP), and anti-β-actin (1:20,000, Biosynthesis, Beijing, China, catalog No. P30002). After washing three times with TBST, membranes were incubated with HRP-conjugated goat anti-mouse or anti-rabbit secondary antibodies (1:10,000, ZSGB-BIO, Beijing, China, catalog Nos. ZB-5305/ZB-2306) at room temperature for 2 h. Protein signals were visualized using an ECL chemiluminescence substrate (Meilun, China, catalog No. MA0186) and detected using a chemiluminescence imaging system (Tanon 5200, Tanon, Shanghai, China). Densitometric analysis was performed using ImageJ software (National Institutes of Health, USA), and relative protein expression was normalized to β-actin.

Immunofluorescence Staining: Cells were seeded onto coverslips in 24-well plates at a density of 2 × 10^4^ cells/well. Cells were fixed with 4% paraformaldehyde (Beyotime, China, catalog No. P0099) for 15 min at room temperature, permeabilized with 0.5% Triton X-100 (Solarbio, Beijing, China, catalog No. T8200) for 10 min, and blocked with 5% BSA (Beyotime, China, catalog No. B885114) for 1 h at room temperature. Primary antibodies (1:100, Proteintech, Wuhan, China; Abcam, Cambridge, UK; Hao Yuan, Shanghai, China) were applied overnight at 4 °C. After three washes with PBS, cells were incubated with FITC- or Cy3-conjugated secondary antibodies (1:200, ZSGB-BIO, China) for 1 h at room temperature in the dark. Nuclei were counterstained with DAPI (Beyotime, China, catalog No. C1005) for 5 min, and coverslips were mounted using an anti-fade mounting medium (Beyotime, China, catalog No. P0126). Images were captured using a fluorescence microscope (ECLIPSE TS2, Nikon, Japan). For quantitative analysis, three independent biological replicates were performed for each experimental condition. For each coverslip, at least five randomly selected fields of view were imaged at 200× or 400× magnification, and a minimum of 100 cells per field were analyzed. The mean fluorescence intensity (MFI) of the target protein was measured using ImageJ software (National Institutes of Health, USA), with background subtraction performed using the region of interest (ROI) from cell-free areas. The relative fluorescence intensity was calculated as the ratio of the MFI of the target signal to the mean DAPI intensity per cell, and the final data were expressed as fold change relative to the control group. All quantifications were performed in a blinded manner to avoid observer bias.

### 2.7. Real-Time Quantitative PCR

Total RNA was extracted from cells using a spin column-based RNA extraction kit (Promega, Madison, WI, USA, catalog No. LS1040), and RNA concentration and purity were assessed using a NanoDrop spectrophotometer (NanoDropOne, Thermo Fisher Scientific, USA). Reverse transcription was performed using 1 μg of total RNA with the PrimeScript RT Reagent Kit (Promega, USA, catalog No. A2790) in a 20-μL reaction volume according to the manufacturer’s instructions. qPCR was performed using SYBR Green Master Mix (Bimake, Houston, TX, USA, catalog No. B21203) on a LightCycler 480 II system (Roche, Basel, Switzerland). Each 10-μL reaction contained 5 μL of 2× SYBR Green Master Mix, 1 μL of cDNA template (diluted 1:5), 0.2 μL each of forward and reverse primers (final concentration 200 nM), and 3.64 μL of DEPC-treated ddH_2_O. The primers used for all target genes were synthesized by Sangon Biotech (Shanghai, China) and are listed in Table 3. The cycling conditions were: initial denaturation at 95 °C for 5 min, followed by 40 cycles of denaturation at 95 °C for 10 s, annealing at 60 °C for 30 s, and extension at 72 °C for 30 s. A melting curve analysis was performed to confirm the specificity of each primer pair. β-Tubulin was used as the internal reference gene, and relative gene expression was calculated using the 2^−ΔΔCt^ method. Each sample was run in triplicate, and each experiment was independently repeated three times.

### 2.8. Histopathology and Immunohistochemistry

Tumor and organ tissues were fixed in 4% paraformaldehyde, embedded in paraffin, and sectioned at 4 μm thickness. Hematoxylin and eosin (HE) staining was performed for morphological evaluation. For immunohistochemistry, sections were deparaffinized, rehydrated, subjected to antigen retrieval in citrate buffer, treated with 3% H_2_O_2_ to block endogenous peroxidase, and blocked with 5% goat serum (Solarbio, China, catalog No. SL038). Sections were incubated with primary antibodies (anti-OGT, 1:200, Proteintech, catalog No. 11576-2-AP; anti-O-GlcNAc, 1:200, Proteintech, catalog No. 65292-1-Ig; anti-Ki-67, 1:200, Abcam; anti-ALDH1, 1:100, Proteintech, catalog No. 15910-1-AP; and anti-CD133, 1:100, Abcam, catalog No. ab19898) overnight at 4 °C, followed by incubation with HRP-conjugated secondary antibodies (1:500, ZSGB-BIO, China) at room temperature for 1 h. Signals were developed using DAB, and sections were counterstained with hematoxylin. Images were captured under a light microscope (ECLIPSE E100, Nikon, Japan).

For quantitative analysis, three independent biological replicates (i.e., tumor samples from three different mice per group) were examined. For each tissue section, at least five randomly selected high-power fields (HPFs, 400× magnification) were imaged, and a minimum of 200 cells per field were evaluated. The staining intensity and distribution were semi-quantitatively assessed using the H-Score method. Briefly, the H-Score was calculated using the formula: H-Score = Σ(*P_i_ × I_i_*), where P_i_ represents the percentage of positive-staining cells (0–100%) and I_i_ represents the staining intensity graded on a scale of 0 (negative), 1 (weak), 2 (moderate), and 3 (strong). For each section, the final H-Score was averaged from five randomly selected HPFs. All immunohistochemical sections were scored independently by two experienced pathologists in a blinded manner. Statistical comparisons of H-Scores between groups were performed using Student’s *t*-test or one-way ANOVA as appropriate.

### 2.9. Special Assays

Alkaline Phosphatase (ALP) Staining: Alkaline phosphatase (ALP) staining was performed using an ALP chromogenic kit (Meilun, China, catalog No. MA0197). Canine osteoblasts were seeded into 12-well plates at a density of 2 × 10^5^ cells/well and cultured until reaching 80–90% confluence. The culture medium was removed, and cells were washed three times with phosphate-buffered saline (PBS, pH 7.4) for 3 min each. Cells were fixed with 4% paraformaldehyde at room temperature for 15 min, followed by three washes with TBST (Tris-buffered saline containing 0.1% Tween-20) for 3 min each. The ALP staining working solution was prepared according to the manufacturer’s instructions (reagents A, B, and C mixed at a 1:1:1 volume ratio). An appropriate volume of the staining solution was added to each well and incubated at room temperature for 30 min in the dark. After staining, cells were washed once with PBS, and images were captured using an inverted microscope (ECLIPSE TS2, Nikon, Japan).

Quantification of UDP-N-acetylglucosamine (UDP-GlcNAc): Intracellular UDP-N-acetylglucosamine (UDP-GlcNAc) levels were quantified using an enzyme-linked immunosorbent assay (ELISA) kit (Jing Kang, Nanjing, China, catalog No. JLC-F0085). Canine osteosarcoma cells cultured under different glucose concentrations were collected and washed twice with ice-cold PBS (0.01 M, pH 7.4). Cell pellets were resuspended in PBS at a ratio of 200 μL per 1 × 10^6^ cells and subjected to sonication on ice (200 W, 3 s pulses with 5 s intervals, repeated 3 times). The lysates were centrifuged at 10,000× *g* for 10 min at 4 °C, and the supernatants were collected for analysis. All procedures were performed according to the manufacturer’s instructions. Briefly, standard wells (with concentrations of 10, 20, 40, 80, 160, and 320 ng/L) and sample wells were set up, and 50 μL of each standard or sample was added to the corresponding wells. Subsequently, 100 μL of horseradish peroxidase (HRP)-conjugated detection antibody was added to each well, followed by incubation at 37 °C for 60 min. After five washes, 50 μL each of chromogenic substrates A and B were added to each well and incubated at 37 °C for 15 min in the dark. The reaction was terminated by adding 50 μL of stop solution, and the optical density (OD) was immediately measured at 450 nm using a microplate reader (SpectraMax iD3, Molecular Devices, USA). A standard curve was constructed by plotting OD values of standards against their concentrations, and UDP-GlcNAc concentrations in samples were calculated by interpolation from the standard curve. Total protein concentration was determined using the BCA method (Meilun, catalog No. MA0082), and UDP-GlcNAc content was expressed as nanograms per milligram of total protein (ng/mg protein). Each sample was run in triplicate, and the experiment was independently repeated three times.

### 2.10. Statistical Analysis

All experiments were independently repeated at least three times, and data are presented as mean ± standard deviation (SD). Statistical analyses were performed using GraphPad Prism 7.0 software. Comparisons between two groups were conducted using Student’s *t*-test, while comparisons among multiple groups were performed using one-way ANOVA followed by Tukey’s post hoc test. A *p*-value < 0.05 was considered statistically significant, and *p* < 0.01 was considered highly significant.

## 3. Results

### 3.1. High Glucose Drives Malignant Phenotypes and Stemness Acquisition in Canine Osteosarcoma Cells

**Figure 1:**
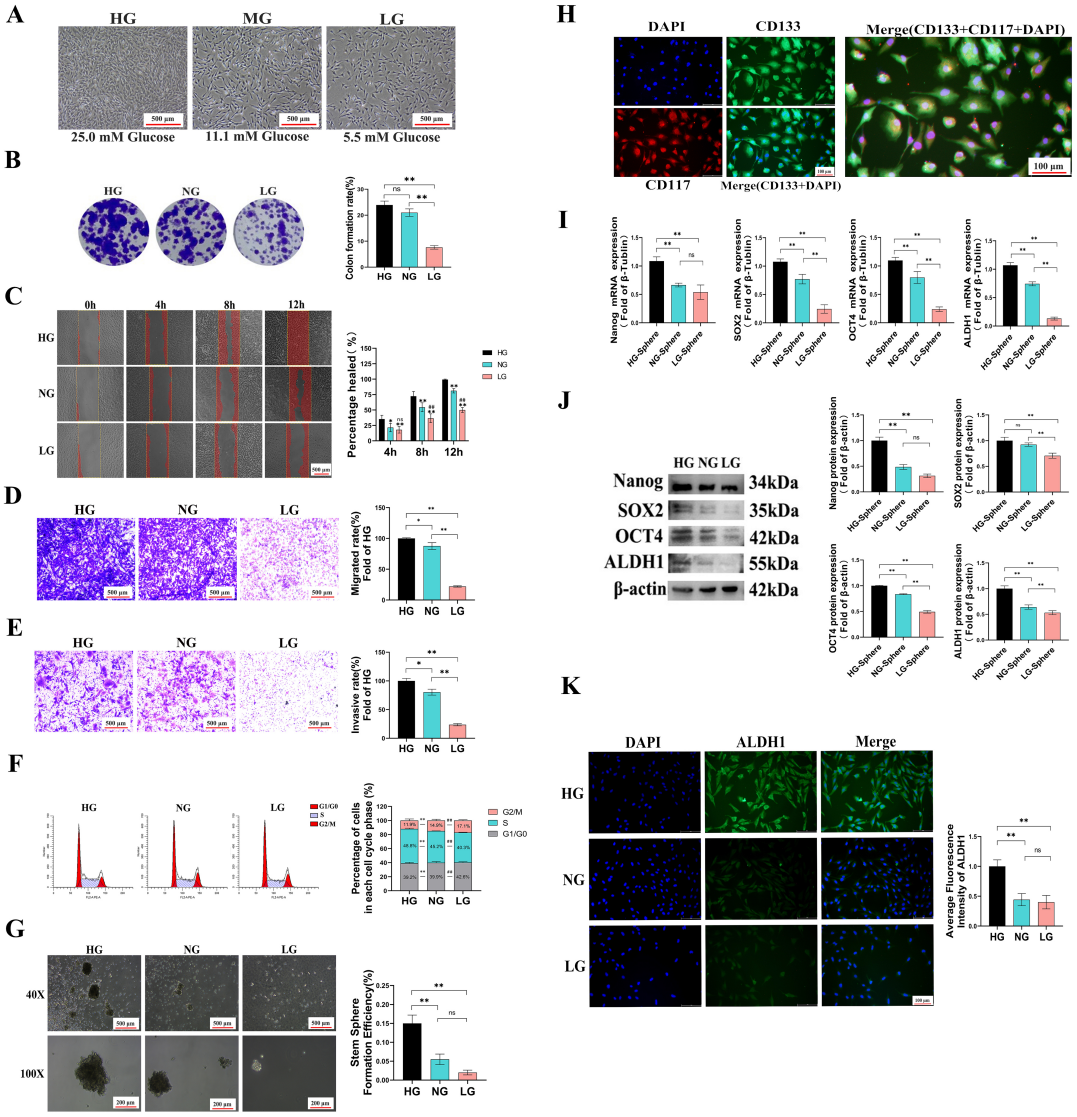
High-glucose microenvironment promotes malignant behaviors and stem-like properties of canine osteosarcoma cells in a dose-dependent manner. (**A**) Representative bright-field morphology images of McKinley cells cultured under HG (25.0 mM), NG (11.1 mM), and LG (5.5 mM) glucose conditions. (**B**) Colony formation staining images and corresponding quantitative analysis of colony numbers. (**C**) Representative images of wound healing assay captured at 0, 4, 8, and 12 h, and statistical histogram of wound closure percentage at each time point. (**D**,**E**) Representative micrographs of Transwell migration (**D**) and invasion (**E**) assays, with quantitative analysis of migrated and invaded cell numbers. (**F**) Flow cytometric analysis of cell cycle distribution, with stacked bar chart showing the proportions of cells in G1, S, and G2/M phases. Red solid stands for G1/G0 phase, blue diagonal stripes represent S phase, and another red solid indicates G2/M phase. ## indicates significant difference between NG and LG groups. (**G**) Representative micrographs of tumor spheres at 40× and 100× magnifications, and statistical analysis of sphere-forming efficiency; scale bar = 500 μm. (**H**) Immunofluorescence co-staining of stemness surface markers CD133 (green) and CD117 (red), with DAPI counterstaining for nuclei and Merge representing colocalization; scale bar = 100 μm. (**I**) qRT-PCR analysis of relative mRNA expression of stemness-related genes NANOG, SOX2, OCT4, and ALDH1 in both adherent cells and tumor spheres. (**J**) Western blot analysis of NANOG, SOX2, OCT4, and ALDH1 protein expression, with β-actin as the loading control; bar graphs show densitometric quantification normalized to β-actin. (**K**) Immunofluorescence staining of ALDH1 in adherent monolayer cells, and quantitative analysis of mean ALDH1 fluorescence intensity. Blue signals represent DAPI nuclear staining, green signals represent ALDH1 immunofluorescence, and Merge panels combine the two channels; scale bar = 100 μm. All data are presented as mean ± SD from three independent biological replicates. * *p* < 0.05, ** *p* < 0.01, ns represents no significant difference. HG, high glucose (25.0 mM); NG, normal glucose (11.1 mM); LG, low glucose (5.5 mM).

To investigate the direct regulatory effects of extracellular glucose concentration on the malignant behaviors and stem-like properties of canine osteosarcoma McKinley cells, three glucose gradient culture conditions were established: high glucose (HG, 25.0 mM), normal physiological glucose (NG, 11.1 mM), and low glucose (LG, 5.5 mM). A comprehensive panel of phenotypic and molecular assays was systematically performed, including cell morphology observation, colony formation, wound healing, Transwell migration and invasion, flow cytometric cell cycle analysis, tumor sphere formation, immunofluorescence staining of stemness markers, qRT-PCR, and Western blot (Figure 1).

Bright-field morphological observation (Figure 1A) revealed marked differences in cell density among the three groups after identical culture durations. The HG group exhibited dense and disorganized spindle-shaped growth with extensive overlapping and stacking. The NG group displayed moderate proliferation with uniform monolayer spreading. In contrast, the LG group showed significantly retarded proliferation, characterized by sparse distribution and elongated cell bodies. These observations preliminarily suggested that high glucose accelerates osteosarcoma cell proliferation.

The colony formation assay (Figure 1B) was performed to evaluate long-term clonogenic potential. After 8 days of continuous culture, the HG group formed the largest number of macroscopic colonies with the greatest individual sizes. The NG group showed a moderate reduction in both colony number and size, whereas the LG group formed only scarce and minute colonies. Quantitative analysis revealed statistically significant differences in colony-forming efficiency among all three groups (*p* < 0.05), confirming that high glucose markedly enhances the long-term clonogenic capacity of osteosarcoma cells.

The wound healing assay was performed to quantitatively assess cell migration at 0, 4, 8, and 12 h post-scratching (Figure 1C). At 4 h, the wound gap in the HG group had already begun to narrow, while the NG and LG groups showed almost no change. At 8 h, intergroup differences became more pronounced, with closure rates of approximately 60% in the HG group, 35% in the NG group, and less than 20% in the LG group. At 12 h, the HG group wounds were nearly completely closed, whereas the NG group achieved only about 65% closure, and the LG group still retained more than half of the original wound area. Wound closure efficiency at all four time points strictly followed the HG > NG > LG gradient, with intergroup differences progressively widening over time. These results confirm that high glucose sustainably accelerates the horizontal migration of adherent osteosarcoma cells, while low glucose persistently suppresses migratory capacity.

Transwell migration and invasion assays further verified the dose-dependent regulation of glucose on vertical cell motility (Figure 1D,E). The HG group exhibited the highest number of cells migrating through the membrane or invading through the Matrigel-coated membrane, followed by the NG group, with the LG group showing the lowest counts. Quantitative analysis revealed statistically significant differences among the three groups for both migration and invasion (*p* < 0.05), consistent with the wound healing results, collectively demonstrating that high glucose simultaneously enhances both the migration capacity and extracellular matrix invasion ability of osteosarcoma cells.

Flow cytometric analysis of cell cycle distribution was performed using PI staining (Figure 1F). The HG group exhibited a significantly increased proportion of cells in S phase, accompanied by a corresponding decrease in G0/G1 phase cells, indicating that abundant glucose promotes G1-to-S phase transition, relieves proliferative arrest, and drives cells into active DNA synthesis and mitotic division. In contrast, the LG group displayed pronounced G0/G1 phase arrest, with a sharp reduction in S phase cells, suggesting that a large population of cells was blocked at the G1/S checkpoint and failed to initiate DNA replication. This indicates that under glucose-deprived conditions, cells actively exit the proliferative cycle and enter a quiescent state. The NG group showed an intermediate cell cycle distribution between the HG and LG groups. The differences in G0/G1 and S phase proportions among the three groups were statistically significant. This gradient pattern demonstrates that glucose concentration determines whether osteosarcoma cells can traverse the G1/S checkpoint: high glucose relieves cell cycle arrest and drives continuous proliferation, whereas low glucose arrests cells at G1 phase, blocks cell cycle progression, and suppresses proliferation.

The tumor sphere formation assay was conducted to evaluate the self-renewal capacity of cancer stem cell subpopulations (Figure 1G). At 40× magnification, the HG group generated abundant large, morphologically intact tumor spheres uniformly distributed across the culture plate. The NG group produced markedly fewer spheres with smaller diameters, while the LG group yielded only scattered, minute, and structurally compromised spheres. At 100× magnification, morphological differences were more pronounced: HG spheres exhibited densely packed cells with smooth, intact edges; NG spheres showed loosely attached peripheral cells; and LG spheres displayed extensive cell detachment and irregular contours. Quantitative analysis revealed a significant positive correlation between sphere-forming efficiency and glucose concentration (*p* < 0.05), confirming that high glucose robustly drives the acquisition of stem-like properties in osteosarcoma cells.

To verify the stem-like identity of the formed tumor spheres, double immunofluorescence staining for CD133 (green) and CD117 (red)—two classical osteosarcoma stem cell surface markers—was performed on HG-derived spheres (Figure 1H). Intense green (CD133) and red (CD117) fluorescence signals were observed throughout the entire sphere structure, and the merged images revealed abundant double-positive cells distributed in both the core and peripheral layers. These results provide direct visual evidence that high glucose-induced tumor spheres possess typical stem-like molecular characteristics.

qRT-PCR and Western blot analyses were performed on RNA and protein extracted from tumor spheres to quantify the expression of the core stemness transcription factors NANOG, SOX2, OCT4, and the functional stemness marker ALDH1 (Figure 1I,J). Both transcriptional and protein levels revealed consistent results, with all four markers exhibiting a stepwise downregulation following the HG > NG > LG gradient. Intergroup differences were statistically significant (* *p* < 0.05, ** *p* < 0.01), confirming at the molecular level that high glucose upregulates stemness-associated gene expression within tumor spheres. All mRNA expression quantification of target genes is summarized in Supplementary Table S1. All band gray value statistics of Western Blot are shown in Supplementary Table S2. All subsequent quantitative data are provided in supplementary tables, so this statement will not be repeated in the following results sections.

Additionally, ALDH1 single-label immunofluorescence staining was performed on adherent monolayer cells (Figure 1K). The HG group exhibited the highest mean fluorescence intensity, followed by the NG group, with the LG group showing the lowest intensity, consistent with the molecular detection results from tumor spheres. Statistical data of immunofluorescence intensity are listed in Table S3. All subsequent quantitative data are provided in supplementary tables, so this statement will not be repeated in the following results sections.

Collectively, phenotypic assays on adherent cells confirmed that high glucose promotes proliferation, colony formation, migration, invasion, and cell cycle progression of osteosarcoma cells. Sphere-specific immunofluorescence and molecular quantification collectively demonstrated that elevated glucose concentrations significantly enhance stem-like properties and upregulate multiple stemness markers, establishing a complete dose-dependent relationship by which the high-glucose microenvironment positively regulates the maintenance of stem-like properties in osteosarcoma.

### 3.2. The Hexosamine Biosynthetic Pathway (HBP) Promotes Stemness in Canine Osteosarcoma Cells

#### 3.2.1. Metabolomics Reveals HBP Activation in Canine Osteosarcoma Cells

**Figure 2:**
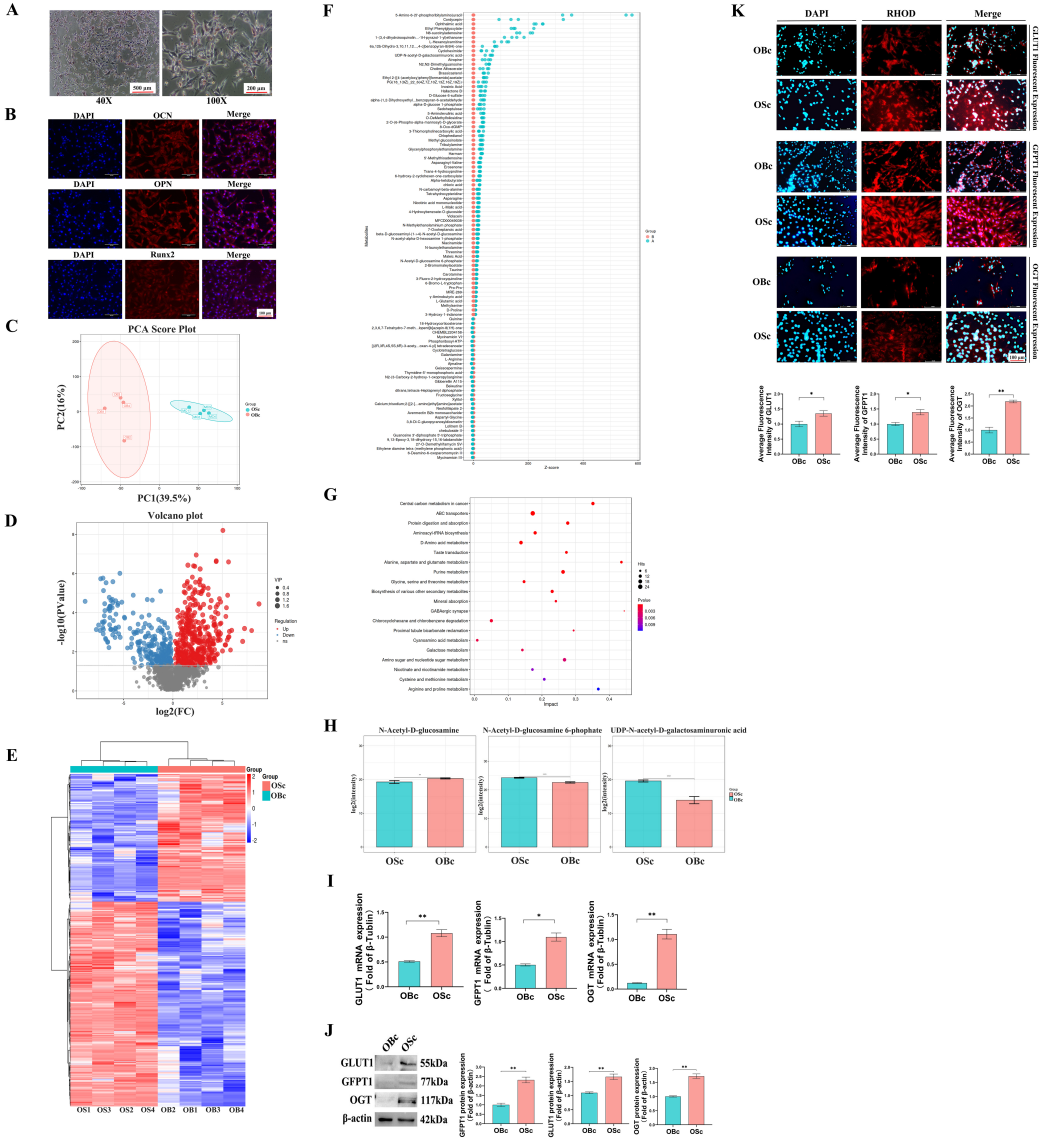
Untargeted metabolomics screening identifies the HBP as the core differential metabolic pathway between normal osteoblasts (OBc) and canine osteosarcoma cells (OSc). (**A**) Representative bright-field images of alkaline phosphatase (ALP) cytochemical staining in OBc osteoblasts, showing 40× and 100× magnifications; scale bar = 100 μm. (**B**) Multi-field immunofluorescence co-staining of osteogenic markers OCN, OPN, and Runx2 in OBc cells; nuclei were counterstained with DAPI (blue), and target proteins were labeled with RHOD (red fluorescence); scale bar = 100 μm. (**C**) PCA score plot of all intracellular metabolites from OBc and OSc cells, with ellipses representing 95% confidence intervals. (**D**) Volcano plot of differentially abundant metabolites: red dots indicate significantly upregulated metabolites in OSc compared with OBc, blue dots indicate significantly downregulated metabolites, and gray dots indicate metabolites with no significant difference; the x-axis represents log_2_(fold change), and the y-axis represents −log_10_(*p*-value). (**E**) Hierarchical clustering heatmap of all significantly differential metabolites; red blocks represent high-abundance metabolites, and blue blocks represent low-abundance metabolites. (**F**) Z-score ranking plot sorted by enrichment significance. Full uncropped high-resolution mass spectrum images are available in Appendix Figure A1. The full names of metabolites abbreviated with ellipses in this plot are listed in the caption of Appendix Figure A1. (**G**) KEGG pathway enrichment bubble plot; bubble size corresponds to the number of enriched differential metabolites, and bubble color represents the enrichment factor. Full uncropped high-resolution mass spectrum images are available in Appendix Figure A2. (**H**) Quantitative bar graph of the relative levels of three core HBP intermediates—N-acetyl-L-glucosamine, N-acetylglucosamine-6-phosphate, and UDP-N-acetylgalactosamine. (**I**) qRT-PCR analysis of GLUT1, GFPT1, and OGT mRNA expression levels in OBc and OSc cells. (**J**) Western blot analysis of GLUT1, GFPT1, and OGT protein expression, with β-actin as the loading control; bar graphs show densitometric quantification normalized to β-actin. (**K**) RHOD fluorescence-based glucose uptake assay images, with DAPI counterstaining for nuclei and Merge representing merged channels, Blue signals stand for DAPI nuclear staining, red signals represent RHOD glucose uptake fluorescence, and the merged panel combines both two channels; the bar graph on the right shows quantification of mean RHOD fluorescence intensity from multiple random fields; scale bar = 100 μm. All data are presented as mean ± SD from three independent biological replicates. All statistical significance markers (* *p* < 0.05, ** *p* < 0.01, **** *p* < 0.0001) in this figure indicate comparisons between the OSc osteosarcoma group and the OBc normal osteoblast group.

To systematically characterize the global metabolic differences between normal osteoblasts (OBc) and canine osteosarcoma cells (OSc), we first performed two independent cell identification assays to validate the osteoblast identity, ensuring the reliability of the cell model for subsequent metabolomic analysis (Figure 2A,B). Alkaline phosphatase (ALP) staining, a hallmark enzymatic marker of mature osteoblasts, revealed extensive dark purple positive precipitates throughout the OBc monolayer, with nearly all cells exhibiting strong positive staining at both 40× and 100× magnifications, preliminarily confirming that the cultured OBc cells possessed intact osteogenic differentiation activity (Figure 2A). Parallel immunofluorescence co-staining of three core osteogenic markers—OCN, OPN, and Runx2—demonstrated uniformly intense red fluorescent signals within the OBc cytoplasm, with no large negative cell populations, as visualized by RHOD fluorescence with DAPI counterstaining for nuclei (Figure 2B). Combined with the ALP staining phenotype, these results collectively confirmed that the OBc cells used in this study were qualified mature normal osteoblasts of sufficient purity, meeting the stringent experimental requirements for subsequent untargeted metabolomic comparison between osteosarcoma and osteoblasts.

Having validated the cell models, we next extracted protein-free metabolites from OBc and OSc cells and performed untargeted metabolomics using liquid chromatography-mass spectrometry (LC-MS) to systematically compare the comprehensive intracellular metabolic profiles of the two cell types (Figure 2C–G). Principal component analysis (PCA) revealed that PC1 and PC2 explained 39.5% and 20.6% of the total variance, respectively, with 95% confidence ellipses completely separating the OBc and OSc biological replicates into two independent non-overlapping regions; the tight clustering of triplicate samples within each group with no outliers indicated stable and significant intrinsic metabolic differences between osteoblasts and osteosarcoma cells, with good data reproducibility (Figure 2C). Volcano plot analysis of differentially abundant metabolites identified numerous significantly altered metabolites: red dots representing metabolites significantly upregulated in OSc compared with OBc, blue dots representing downregulated metabolites, and gray dots representing metabolites with no statistical difference (Figure 2D). Notably, a substantial number of hexose derivatives, hexosamine precursors, hexose phosphates, and UDP-sugar intermediates were enriched in the upregulated red cluster, preliminarily suggesting aberrant activation of glucose metabolism and the hexosamine biosynthetic pathway in osteosarcoma cells. Hierarchical clustering heatmap of all significantly differential metabolites revealed two major clusters: the first cluster showed high accumulation in OBc and low expression in OSc, while the second cluster exhibited the opposite trend, with abundant enrichment in OSc cells (Figure 2E).

To further stratify metabolite abundance patterns, we performed Z-score ranking analysis, which clearly demonstrated that in the high Z-score range (200 to 600), the cyan OSc dots for all metabolites were markedly shifted to the right, indicating substantial accumulation of these small molecules in osteosarcoma cells (Figure 2F). Among the top-ranking metabolites were numerous HBP-specific intermediates, including UDP-N-acetyl-D-galactosaminuric acid, N-acetyl-D-glucosamine 6-phosphate, beta-D-glucosaminyl-(1→4)-N-acetyl-D-glucosamine, and N-acetyl-alpha-D-hexosamine 1-phosphate, visually demonstrating the specific enrichment of hexosamine synthesis precursors and UDP-sugar derivatives in osteosarcoma cells. In contrast, in the low Z-score range, the red OBc dots were generally shifted to the right, corresponding to amino acids, organic acids, and lipid-related metabolites with higher expression levels in normal osteoblasts. The complete separation of metabolite expression patterns between the two cell types further demonstrated that OSc osteosarcoma cells had undergone glucose metabolic reprogramming, with overall significant upregulation of HBP pathway-related metabolites.

KEGG pathway enrichment analysis revealed that “Amino sugar and nucleotide sugar metabolism” (i.e., the HBP hexosamine pathway) appeared as the most significantly enriched pathway, with deep red bubble color indicating high statistical significance, and both the impact factor and the number of enriched metabolites ranking at the forefront among all pathways (Figure 2G). Other highly enriched pathways included “Central carbon metabolism in cancer,” “ABC transporters,” “Protein digestion and absorption,” “Purine metabolism,” and “Alanine, aspartate, and glutamate metabolism.” Compared with other amino acid, lipid, and xenobiotic degradation pathways, the HBP exhibited substantially greater enrichment significance and scale. Combined with the Z-score ranking plot showing specific high expression of numerous HBP intermediates in OSc osteosarcoma cells, these two analyses mutually corroborated each other, collectively demonstrating from both single-metabolite abundance and overall pathway enrichment dimensions that the hexosamine biosynthetic pathway (HBP) is the most central and differentially prominent metabolic pathway between normal osteoblasts and canine osteosarcoma cells, serving as the key target for subsequent mechanistic investigation.

To provide direct quantitative evidence of HBP hyperactivation, we performed targeted quantification of three representative core HBP intermediates: N-acetyl-L-glucosamine, N-acetylglucosamine-6-phosphate, and UDP-N-acetylgalactosamine. All three HBP intermediates showed significantly higher relative levels in OSc osteosarcoma cells compared with OBc osteoblasts, directly confirming at the metabolite substrate level that the HBP synthetic cascade is continuously hyperactivated in canine osteosarcoma cells (Figure 2H). To further characterize the expression differences in key functional molecules regulating HBP activity, we performed multiple assays in parallel, including transcriptional, translational, and immunofluorescence detection, targeting the glucose transporter and two core HBP enzymes (Figure 2I–K). qRT-PCR analysis revealed that the mRNA expression levels of GLUT1, GFPT1, and OGT were all significantly elevated in OSc compared with OBc, indicating that osteosarcoma cells coordinately upregulated the entire upstream molecular cascade required for HBP substrate supply and glycosylation modification at the transcriptional level (Figure 2I). Western blot analysis confirmed these findings at the protein level, demonstrating markedly higher abundances of GLUT1, GFPT1, and OGT proteins in OSc than in OBc, with densitometric quantification revealing statistically significant differences between the two groups, fully consistent with the transcriptional trends (Figure 2J). To further assess functional glucose uptake capacity, we performed RHOD fluorescence-based glucose uptake assay. OSc monolayer cells exhibited dense and widespread red fluorescence signals, whereas OBc cells showed only sparse and weak signals; quantitative analysis of mean fluorescence intensity confirmed that OSc cells possessed significantly greater glucose uptake capacity than OBc, continuously supplying sufficient carbon substrates for intracellular HBP pathway activation (Figure 2K).

Collectively, the comprehensive series of results—including osteoblast identity validation, untargeted LC-MS metabolomic global screening, targeted quantification of HBP intermediates, multi-level transcriptional/protein expression profiling of HBP core molecules, and glucose uptake fluorescence functional assays—collectively demonstrated that canine osteosarcoma cells undergo profound global metabolic reprogramming compared with normal osteoblasts. Among all altered metabolic pathways, the hexosamine biosynthetic pathway (HBP) exhibited the most significant aberrant hyperactivation, accompanied by enhanced glucose uptake capacity, substantial accumulation of HBP synthesis intermediates, and coordinated upregulation of GLUT1, GFPT1, and OGT at both the mRNA and protein levels. These findings lay a solid foundation for subsequent mechanistic investigations into the regulation of osteosarcoma malignant progression and stemness properties via the HBP/O-GlcNAc axis. All statistical significance markers in this figure (* *p* < 0.05, ** *p* < 0.01) indicate comparisons between the OSc osteosarcoma group and the OBc normal osteoblast group.

#### 3.2.2. Glucose Concentration Activates the HBP/O-GlcNAc Pathway in a Dose-Dependent Manner

**Figure 3:**
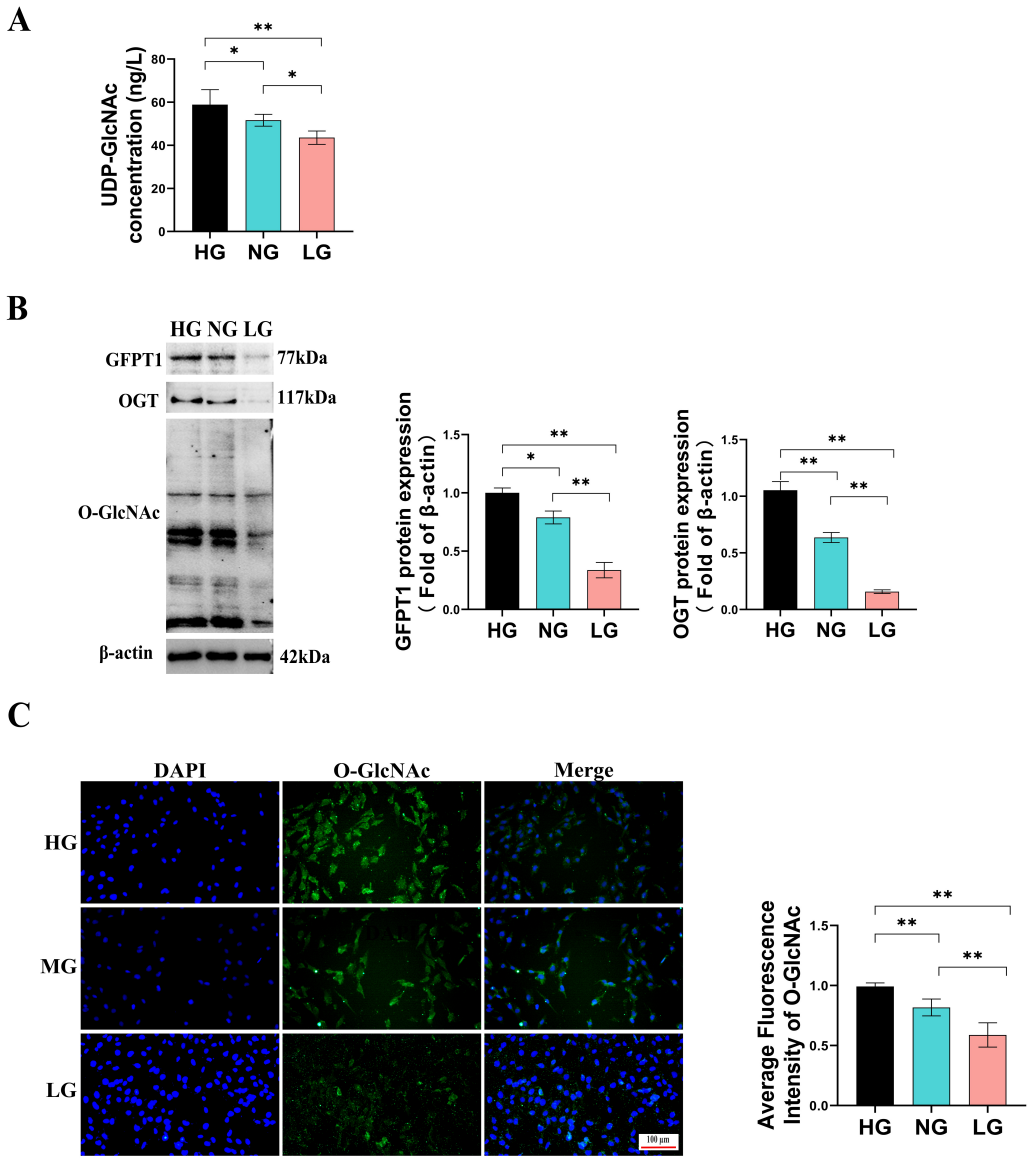
Glucose concentration positively regulates HBP/O-GlcNAc pathway activity in a dose-dependent manner. (**A**) Quantification of intracellular UDP-GlcNAc concentrations in canine osteosarcoma cells cultured under high glucose (HG, 25.0 mM), normal glucose (NG, 11.1 mM), and low glucose (LG, 5.5 mM) conditions (*n* = 3 biological replicates). (**B**) Western blot analysis of the protein expression levels of GFPT1 (the HBP rate-limiting enzyme), OGT (the O-GlcNAc transferase), and global O-GlcNAcylation, with β-actin serving as the loading control. Bar graphs on the right show the densitometric quantification of GFPT1 and OGT normalized to β-actin. (**C**) Immunofluorescence staining of global O-GlcNAc modification in cells treated with the three glucose gradients. Blue signals indicate DAPI-stained cell nuclei, green signals represent O-GlcNAc modification immunofluorescence, and merged panels combine both two channels. The bar graph on the right represents the quantification of mean O-GlcNAc fluorescence intensity (scale bar = 100 μm). All data are presented as mean ± standard deviation (SD). * *p* < 0.05, ** *p* < 0.01. HG, high glucose; NG, normal glucose; LG, low glucose.

The untargeted metabolomics screening comparing normal osteoblasts (OBc) with canine osteosarcoma cells (OSc) identified the amino sugar and nucleotide sugar metabolic pathway (Hexosamine Biosynthetic Pathway, the HBP/O-GlcNAc pathway) as a candidate metabolic axis that is significantly and aberrantly activated in osteosarcoma cells. To verify whether extracellular glucose concentration directly regulates the activity of this pathway, canine osteosarcoma cells were cultured under three glucose gradients: high glucose (HG, 25.0 mM), normal glucose (NG, 11.1 mM), and low glucose (LG, 5.5 mM). The levels of the core HBP metabolite, key enzymatic proteins, and global O-GlcNAcylation were systematically examined (Figure 3).

ELISA analysis revealed that the intracellular content of UDP-GlcNAc, the rate-limiting substrate of the HBP, decreased in a gradient manner in parallel with glucose concentration (Figure 3A). The UDP-GlcNAc level in the HG group was significantly higher than that in both the NG and LG groups, with statistically significant differences between any two groups (* *p* < 0.05, ** *p* < 0.01), providing metabolite-level evidence that a high-glucose environment promotes the accumulation of the core HBP precursor.

Western blot analysis further confirmed the dose-dependent regulation of HBP proteins by glucose (Figure 3B). The protein expression levels of GFPT1 (the rate-limiting enzyme of the HBP pathway) and OGT (the sole transferase mediating O-GlcNAc modification) were synchronously downregulated as glucose concentration decreased from HG to LG. Concurrently, global O-GlcNAcylation levels exhibited an identical gradient decline. Densitometric quantification normalized to β-actin revealed statistically significant differences among the three groups in GFPT1 and OGT protein expression, as well as in global O-GlcNAcylation levels.

Immunofluorescence staining was performed to visualize the intracellular distribution and intensity of O-GlcNAc modification in situ (Figure 3C). Cells in the HG group displayed the strongest green fluorescence signal (representing O-GlcNAc modification), with successively weaker signals in the NG and LG groups. Quantitative analysis of mean fluorescence intensity was highly consistent with the UDP-GlcNAc quantification and Western blot results, further confirming that high glucose significantly upregulates global protein O-GlcNAcylation.

Collectively, the glucose gradient intervention experiments established a positive dose-dependent regulatory relationship between the high-glucose microenvironment and HBP/O-GlcNAc pathway activity, thereby validating the hypothesis generated from the OBc/OSc metabolomics screening. All subsequent functional experiments in this study—including stemness phenotype characterization, gene knockdown rescue assays, and in vivo xenograft tumor formation in nude mice—were uniformly conducted under HG (25.0 mM) culture conditions, aiming to further elucidate the molecular mechanisms by which the HBP, activated in the high-glucose microenvironment, mediates malignant stemness phenotypes in osteosarcoma.

#### 3.2.3. GFPT1 Silencing Suppresses Malignant Behaviors and Stemness in Canine Osteosarcoma Cells

**Figure 4:**
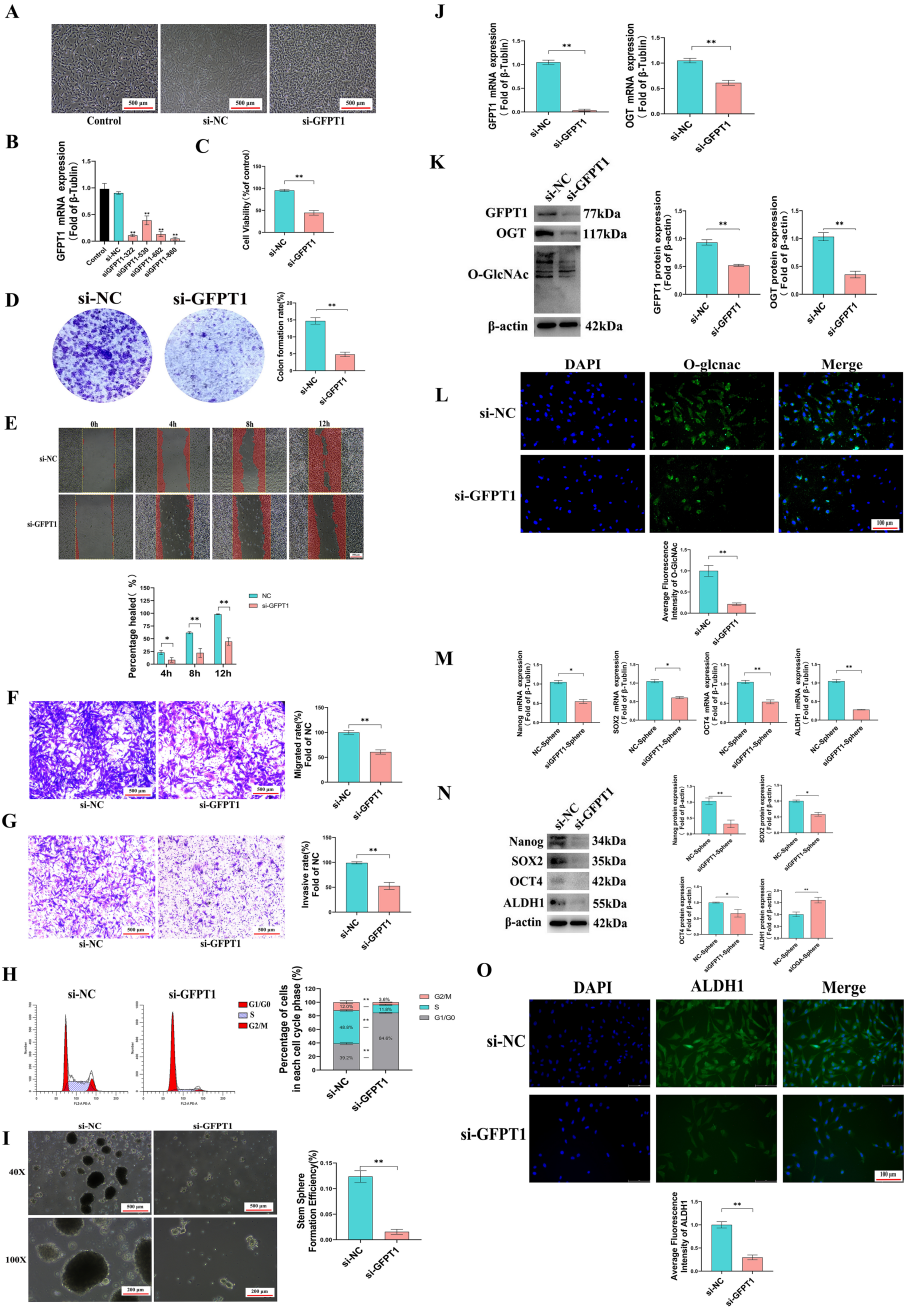
Transient silencing of GFPT1 suppresses HBP/O-GlcNAc pathway activity and attenuates malignant behaviors and stemness properties in canine osteosarcoma cells under high-glucose conditions. (**A**) Representative bright-field images of McKinley cells transfected with si-NC or si-GFPT1 under high-glucose conditions; scale bar = 100 μm. (**B**) qRT-PCR analysis of GFPT1 silencing efficiency in adherent cells. (**C**) CCK-8 assay measuring relative cell viability between si-NC and si-GFPT1 groups. (**D**) Representative images of crystal violet-stained colonies and quantitative bar graph of colony numbers. (**E**) Representative images of wound healing assay at 0, 4, 8, and 12 h, and quantitative bar graph of wound closure rates at each time point. (**F**,**G**) Representative images of crystal violet-stained Transwell migration (**F**) and invasion (**G**) assays, and quantitative analysis of migrated and invaded cell numbers. (**H**) Flow cytometric analysis of cell cycle distribution, with stacked bar graph showing the percentages of cells in G0/G1, S, and G2/M phases. Red solid stands for G1/G0 phase, blue diagonal stripes represent S phase, and another red solid indicates G2/M phase. (**I**) Representative images of tumor spheres at 40× and 100× magnifications, and quantitative analysis of sphere-forming efficiency; scale bar = 500 μm. (**J**) qRT-PCR analysis of GFPT1 and OGT mRNA expression levels in adherent cells. (**K**) Western blot analysis of GFPT1, OGT, and global O-GlcNAcylation protein levels, with β-actin as the loading control; bar graphs show densitometric quantification of GFPT1 and OGT normalized to β-actin. (**L**) Immunofluorescence staining of global O-GlcNAcylation in adherent cells, with DAPI counterstaining for nuclei and Merge representing merged channels. Blue signals indicate DAPI-stained cell nuclei, green signals represent O-GlcNAc modification immunofluorescence, and merged panels combine both two channels. Bar graph shows quantification of mean O-GlcNAc fluorescence intensity; scale bar = 100 μm. (**M**) qRT-PCR analysis of stemness-associated genes NANOG, SOX2, OCT4, and ALDH1 mRNA expression in tumor spheres from si-NC and si-GFPT1 groups. (**N**) Western blot analysis of stemness protein expression in tumor spheres, with β-actin as the loading control; bar graphs show densitometric quantification of target proteins normalized to β-actin. (**O**) Immunofluorescence staining of ALDH1 in adherent monolayer cells, and quantitative analysis of mean ALDH1 fluorescence intensity. Blue signals represent DAPI nuclear staining, green signals represent ALDH1 immunofluorescence, and Merge panels combine the two channels; scale bar = 100 μm. All data are presented as mean ± SD from three independent biological replicates. All statistical significance markers (* *p* < 0.05, ** *p* < 0.01) indicate comparisons between the si-GFPT1 group and the si-NC-negative control group.

To verify whether HBP activation is a critical and indispensable mediator through which the high-glucose microenvironment regulates malignant phenotypes and stemness properties in osteosarcoma, transient transfection with siRNA targeting GFPT1, the rate-limiting enzyme of the HBP pathway, was employed to establish a knockdown model, with si-NC serving as the negative control. All cellular functional and molecular assays were uniformly conducted under high-glucose (25.0 mM) conditions to investigate the impact of HBP pathway inhibition on the biological behaviors of canine osteosarcoma McKinley cells (Figure 4).

Bright-field morphological observation (Figure 4A) revealed that si-NC cells exhibited dense growth with robust spindle-shaped proliferation, whereas si-GFPT1 cells displayed sparse spreading, retarded growth, and shrunken cell bodies, preliminarily suggesting that GFPT1 silencing impairs the proliferative capacity of osteosarcoma cells.

The knockdown efficiency of si-GFPT1 was first validated by qRT-PCR (Figure 4B). Compared with the si-NC group, the si-GFPT1 group exhibited a significant reduction in GFPT1 mRNA expression, confirming successful transient knockdown. Among the siGFPT1 sequences tested, siGFPT1-860 showed the highest silencing efficiency and was therefore selected for subsequent experiments. Concurrent qRT-PCR analysis (Figure 4J) revealed that GFPT1 silencing also significantly reduced the mRNA expression of OGT, the core catalytic enzyme mediating O-GlcNAc modification.

Western blot analysis further confirmed the transcriptional findings at the protein level (Figure 4K). GFPT1 silencing markedly reduced the protein abundance of both GFPT1 and OGT, accompanied by a pronounced decrease in global O-GlcNAcylation levels. Densitometric quantification confirmed statistically significant differences in GFPT1 and OGT protein expression between the si-GFPT1 and si-NC groups. Concurrently, immunofluorescence staining for O-GlcNAc modification was performed on adherent cells to visually assess changes in modification levels following HBP inhibition (Figure 4L). The si-NC group displayed intense green fluorescence (representing O-GlcNAc modification), whereas the si-GFPT1 group showed substantially attenuated fluorescence intensity. Quantitative analysis of mean fluorescence intensity confirmed a significant reduction in O-GlcNAc modification signals upon GFPT1 silencing, which was fully consistent with the Western blot results.

CCK-8 assay was performed to quantitatively assess cell proliferation activity (Figure 4C). At all time points examined, the relative cell viability of the si-GFPT1 group was significantly lower than that of the si-NC group, demonstrating that GFPT1 silencing suppresses the proliferative activity of osteosarcoma cells. Colony formation assay was conducted to evaluate long-term clonogenic survival potential (Figure 4D). After continuous culture, the si-NC group formed abundant large, densely packed colonies, whereas the si-GFPT1 group produced significantly fewer colonies with markedly reduced individual sizes. Quantitative analysis confirmed a significant decrease in colony-forming efficiency upon GFPT1 silencing compared with the si-NC group.

Wound healing assay was performed to quantify horizontal cell migration by recording the remaining wound area at 0, 4, 8, and 12 h post-scratching (Figure 4E). At all four time points, the wound closure rate of the si-GFPT1 group was consistently lower than that of the si-NC group, with the migratory gap between the two groups progressively widening over time. At 4 h, si-NC cells had already migrated noticeably toward the wound center, while the si-GFPT1 group showed almost no wound narrowing. At 8 h, the si-NC group achieved approximately 50% closure; at 12 h, the si-NC wounds were nearly completely closed, whereas the si-GFPT1 group reached only about 30% closure. These results demonstrate that GFPT1 silencing severely impairs horizontal migration of osteosarcoma cells. Statistical significance at each time point (* *p* < 0.05, ** *p* < 0.01) was determined relative to the si-NC-negative control group.

Transwell migration and invasion assays were performed using uncoated and Matrigel-coated chambers, respectively, to assess vertical migration and basement membrane invasion (Figure 4F,G). Following 24 h of serum-stimulated incubation and crystal violet staining, the number of migrated and invaded cells in the si-GFPT1 group was markedly reduced compared with the si-NC group. Quantitative analysis confirmed statistically significant differences between the two groups, consistent with the wound healing results, collectively demonstrating that HBP inhibition via GFPT1 silencing simultaneously attenuates both migration and invasion capacities of osteosarcoma cells.

Flow cytometric analysis with PI staining was performed to examine cell cycle distribution following GFPT1 silencing (Figure 4H). Compared with the si-NC group, the si-GFPT1 group exhibited pronounced G0/G1 phase arrest, accompanied by a significant reduction in S and G2/M phase populations. The stacked bar chart visually depicts this cell cycle redistribution, indicating that GFPT1 silencing blocks G1-to-S phase transition and thereby suppresses cell division and proliferation.

Tumor sphere formation assay was performed under ultra-low attachment conditions to enrich for stem cell subpopulations in a high-glucose microenvironment (Figure 4I). At 40× and 100× magnifications, the si-NC group formed large, structurally compact, and morphologically intact tumor spheres, whereas the si-GFPT1 group generated only minute, structurally fragmented microspheres with loosely detached peripheral cells. Quantitative analysis of sphere-forming efficiency confirmed that GFPT1 silencing significantly impaired the self-renewal and enrichment capacity of stem-like tumor cells, with statistically significant differences compared with the si-NC group.

Total RNA and protein were extracted from tumor spheres of both groups to examine the transcriptional and protein expression levels of the core stemness markers NANOG, SOX2, OCT4, and ALDH1. qRT-PCR results (Figure 4M) demonstrated that the mRNA abundances of all four stemness genes were significantly downregulated in si-GFPT1 spheres compared with si-NC spheres. Western blot analysis (Figure 4N) revealed fully consistent trends: GFPT1 silencing markedly reduced the relative protein expression of NANOG, SOX2, OCT4, and ALDH1, with statistically significant differences for each marker compared with the si-NC group.

ALDH1 single-label immunofluorescence staining was performed on adherent monolayer cells to complement the assessment of stemness phenotypes (Figure 4O). The si-NC group exhibited intense green ALDH1 fluorescence signals, whereas the si-GFPT1 group showed markedly diminished fluorescence intensity. Quantitative analysis of mean fluorescence intensity confirmed that GFPT1 silencing reduces the abundance of ALDH1-positive stem-like cell subpopulations, corroborating the molecular findings from tumor spheres.

Collectively, transient silencing of GFPT1, the rate-limiting enzyme of the HBP, effectively blocked the entire HBP/O-GlcNAc signaling axis, as evidenced by downregulated GFPT1 and OGT expression and reduced global O-GlcNAcylation levels. A series of functional assays demonstrated that GFPT1 silencing significantly suppresses proliferation, clonogenic survival, horizontal migration, matrix invasion, cell cycle progression, and cancer stem cell self-renewal in canine osteosarcoma cells under high-glucose conditions. Concurrently, the classical stemness markers NANOG, SOX2, OCT4, and ALDH1 were substantially downregulated at both transcriptional and protein levels. All statistical comparisons in this figure were performed relative to the si-NC-negative control group. Collectively, these results demonstrate that an intact HBP pathway is essential for high-glucose-driven malignant progression and maintenance of stemness properties in osteosarcoma.

### 3.3. In Vitro Validation of O-GlcNAcylation in Regulating Stemness of Canine Osteosarcoma Cells

#### 3.3.1. OGT Knockdown Suppresses Malignant Behaviors and Stemness in Canine Osteosarcoma Cells

**Figure 5:**
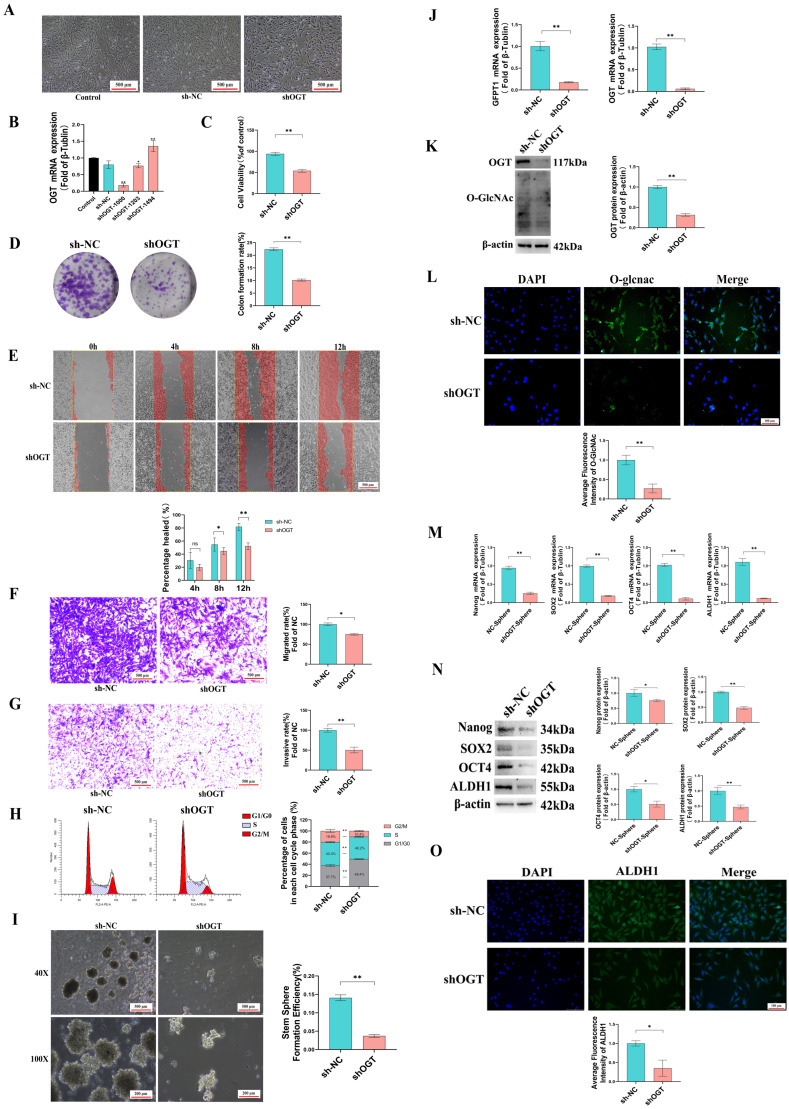
Lentivirus-mediated stable OGT knockdown suppresses global O-GlcNAcylation and blocks high-glucose-induced malignant behaviors and stem-like properties in canine osteosarcoma cells. (**A**) Representative bright-field images of sh-NC and sh-OGT stable cell lines cultured under high-glucose (25.0 mM) conditions; scale bar = 100 μm. (**B**) qRT-PCR analysis of OGT stable knockdown efficiency in adherent cells. (**C**) CCK-8 assay measuring relative cell viability between sh-NC and sh-OGT groups. (**D**) Representative images of crystal violet-stained colonies and quantitative bar graph of colony numbers. (**E**) Representative images of wound healing assay at 0, 4, 8, and 12 h, and quantitative bar graph of wound closure rates at each time point. (**F**,**G**) Representative images of crystal violet-stained Transwell migration (**F**) and invasion (**G**) assays, and quantitative analysis of migrated and invaded cell numbers. (**H**) Flow cytometric analysis of cell cycle distribution, with stacked bar graph showing the percentages of cells in G0/G1, S, and G2/M phases. Red solid stands for G1/G0 phase, blue diagonal stripes represent S phase, and another red solid indicates G2/M phase. (**I**) Representative images of tumor spheres at 40× and 100× magnifications, and quantitative analysis of sphere-forming efficiency; scale bar = 500 μm. (**J**) qRT-PCR analysis of GFPT1 and OGT mRNA expression levels in adherent monolayer cells. (**K**) Western blot analysis of OGT and global O-GlcNAcylation protein levels, with β-actin as the loading control; bar graph shows densitometric quantification of OGT normalized to β-actin. (**L**) Immunofluorescence staining of global O-GlcNAcylation in adherent cells Blue signals indicate DAPI-stained cell nuclei, green signals represent O-GlcNAc modification immunofluorescence, and merged panels combine both two channels. Bar graph shows quantification of mean O-GlcNAc fluorescence intensity; scale bar = 100 μm. (**M**) qRT-PCR analysis of stemness-associated genes NANOG, SOX2, OCT4, and ALDH1 mRNA expression in tumor spheres from sh-NC and sh-OGT groups. (**N**) Western blot analysis of stemness protein expression in tumor spheres, with β-actin as the loading control; bar graphs show densitometric quantification of target proteins normalized to β-actin. (**O**) Immunofluorescence staining of ALDH1 in adherent monolayer cells, and quantitative analysis of mean ALDH1 fluorescence intensity. Blue signals represent DAPI nuclear staining, green signals represent ALDH1 immunofluorescence, and Merge panels combine the two channels; scale bar = 100 μm. All data are presented as mean ± SD from three independent biological replicates. All statistical significance markers (* *p* < 0.05, ** *p* < 0.01) indicate comparisons between the sh-OGT stable knockdown group and the sh-NC lentiviral negative control group, ns represents no significant difference.

The aforementioned transient silencing of GFPT1 confirmed that inhibition of the HBP attenuates the malignant phenotypes of canine osteosarcoma cells. To further verify whether O-GlcNAc modification, as the downstream effector of the HBP pathway, is indispensable for maintaining high-glucose-induced malignant and stemness phenotypes in osteosarcoma, a stable OGT-knockdown cell line (sh-OGT) was established via lentiviral transduction, with sh-NC serving as the negative control. All cellular functional and molecular assays were uniformly conducted under high-glucose (25.0 mM) conditions to investigate the impact of sustained O-GlcNAc modification inhibition on the biological behaviors of osteosarcoma cells (Figure 5).

Bright-field morphological observation (Figure 5A) revealed a marked difference in proliferation between the two groups: sh-NC cells exhibited dense growth with extensive overlapping and aggregation of spindle-shaped cells, whereas sh-OGT cells displayed sparse spreading, retarded proliferation, and shrunken cell bodies, preliminarily suggesting that sustained OGT knockdown suppresses the proliferative capacity of osteosarcoma cells.

The knockdown efficiency of lentivirus-mediated sh-OGT was first validated by qRT-PCR (Figure 5B). Compared with the sh-NC group, both shOGT-1000 and shOGT-1203 significantly reduced OGT mRNA expression, with shOGT-1000 showing substantially higher knockdown efficiency; therefore, shOGT-1000 was selected for subsequent experiments. In contrast, shOGT-1494 unexpectedly upregulated OGT mRNA expression and was excluded from further analysis. Concurrent qRT-PCR analysis (Figure 5J) revealed that stable OGT silencing also significantly reduced the mRNA expression of GFPT1, the upstream rate-limiting enzyme of the HBP, suggesting the existence of a feedback inhibitory loop within the entire HBP.

Western blot analysis further confirmed the transcriptional findings at the protein level (Figure 5K). Stable OGT knockdown markedly reduced OGT protein abundance, accompanied by a pronounced global decrease in O-GlcNAcylation levels. Densitometric quantification confirmed statistically significant differences in both OGT protein expression and global O-GlcNAcylation levels between the sh-OGT and sh-NC groups. Immunofluorescence staining for O-GlcNAc modification was performed on adherent monolayer cells to visually assess changes in modification levels following OGT inhibition (Figure 5L). The sh-NC group displayed widespread and intense green fluorescence (representing O-GlcNAc modification), whereas the sh-OGT group showed substantially attenuated fluorescence intensity throughout the cells. Quantitative analysis of mean fluorescence intensity confirmed a significant reduction in O-GlcNAc modification signals upon stable OGT knockdown, which was fully consistent with the Western blot results.

CCK-8 assay was performed to quantitatively assess cell proliferation activity (Figure 5C). At all time points examined, the relative cell viability of the sh-OGT group was significantly lower than that of the sh-NC group, demonstrating that stable OGT knockdown sustainably suppresses the proliferative activity of osteosarcoma cells under high-glucose conditions.

Colony formation assay was conducted to evaluate long-term clonogenic survival potential (Figure 5D). After static continuous culture, the sh-NC group formed abundant large, densely packed macroscopic colonies, whereas the sh-OGT group produced significantly fewer colonies, which were minute and exhibited loosely organized internal structures. Quantitative analysis confirmed a significant decrease in colony-forming efficiency upon OGT knockdown compared with the sh-NC group.

Wound healing assay was performed to quantify horizontal cell migration by recording the remaining wound area at 0, 4, 8, and 12 h post-scratching (Figure 5E). At all four time points, the wound closure rate of the sh-OGT group was consistently lower than that of the sh-NC group, with the migratory gap between the two groups progressively widening over time. At 4 h, sh-NC cells had already migrated noticeably toward the wound center, while the sh-OGT group showed almost no wound narrowing. At 8 h, the sh-NC group achieved approximately 50% closure; at 12 h, the sh-NC wounds reached nearly 90% closure, whereas the sh-OGT group achieved only about 20% closure. Statistical significance at each time point (* *p* < 0.05, ** *p* < 0.01) was determined relative to the sh-NC-negative control group.

Transwell migration and invasion assays were performed to assess unhindered vertical migration and basement membrane invasion, respectively (Figure 5F,G). Following 24 h of serum-gradient chemotactic incubation and crystal violet staining, cell counts in 200× fields revealed that the number of migrated and invaded cells in the sh-OGT group was markedly reduced compared with the sh-NC group. Quantitative analysis confirmed statistically significant differences between the two groups, consistent with the wound healing results, collectively demonstrating that stable OGT knockdown-mediated suppression of O-GlcNAc modification simultaneously attenuates both horizontal migration and extracellular matrix invasion of osteosarcoma cells.

Flow cytometric analysis with PI staining was performed to examine cell cycle distribution following stable OGT knockdown (Figure 5H). Compared with the sh-NC group, the sh-OGT group exhibited pronounced G0/G1 phase arrest, accompanied by a significant reduction in S and G2/M phase populations. The stacked bar chart visually depicts this cell cycle arrest, indicating that sustained OGT knockdown blocks G1-to-S phase transition and thereby suppresses cell division.

Tumor sphere formation assay was performed under ultra-low attachment conditions to enrich for stem cell subpopulations in a high-glucose microenvironment (Figure 5I). At 40× and 100× magnifications, the sh-NC group formed abundant large, structurally intact tumor spheres with smooth and compact edges, whereas the sh-OGT group generated only scattered, structurally fragmented microspheres with extensively detached peripheral cells. Quantitative analysis of sphere-forming efficiency confirmed that stable OGT silencing significantly impairs the self-renewal and enrichment capacity of stem-like tumor cells, with statistically significant differences compared with the sh-NC group.

Total RNA and protein were extracted from tumor spheres of both groups to examine the mRNA and protein expression levels of the core stemness transcription factors NANOG, SOX2, OCT4, and the functional stemness marker ALDH1. qRT-PCR results (Figure 5M) demonstrated that the mRNA abundances of all four stemness genes were significantly downregulated in sh-OGT spheres compared with sh-NC spheres. Western blot analysis (Figure 5N) revealed fully consistent trends: stable OGT silencing markedly reduced the relative protein expression of NANOG, SOX2, OCT4, and ALDH1, with statistically significant differences for each marker compared with the sh-NC group.

ALDH1 single-label immunofluorescence staining was performed on adherent monolayer cells as a complementary assessment of stemness phenotypes (Figure 5O). The sh-NC group exhibited uniform and intense green ALDH1 fluorescence signals, whereas the sh-OGT group showed markedly diminished fluorescence intensity. Quantitative analysis of mean fluorescence intensity confirmed that stable OGT knockdown reduces the abundance of ALDH1-positive stem-like cell subpopulations, corroborating the molecular findings from tumor spheres.

Collectively, lentivirus-mediated stable OGT knockdown sustainably suppressed global O-GlcNAc modification and concurrently downregulated upstream GFPT1 expression through a feedback inhibitory loop, thereby blocking the entire HBP/O-GlcNAc signaling axis. A comprehensive series of functional assays demonstrated that stable OGT silencing significantly suppresses proliferation, long-term clonogenic survival, horizontal migration, matrix invasion, cell cycle progression, and cancer stem cell self-renewal in canine osteosarcoma cells under high-glucose conditions. Concurrently, the classical stemness markers NANOG, SOX2, OCT4, and ALDH1 were substantially downregulated at both transcriptional and protein levels. All statistical comparisons in this figure were performed relative to the sh-NC-negative control group. Collectively, these results demonstrate that OGT-mediated O-GlcNAc modification is an indispensable downstream effector pathway through which high glucose drives malignant progression and maintains stemness in osteosarcoma.

#### 3.3.2. OGA Silencing Enhances Malignant Behaviors and Stemness in Canine Osteosarcoma Cells

**Figure 6:**
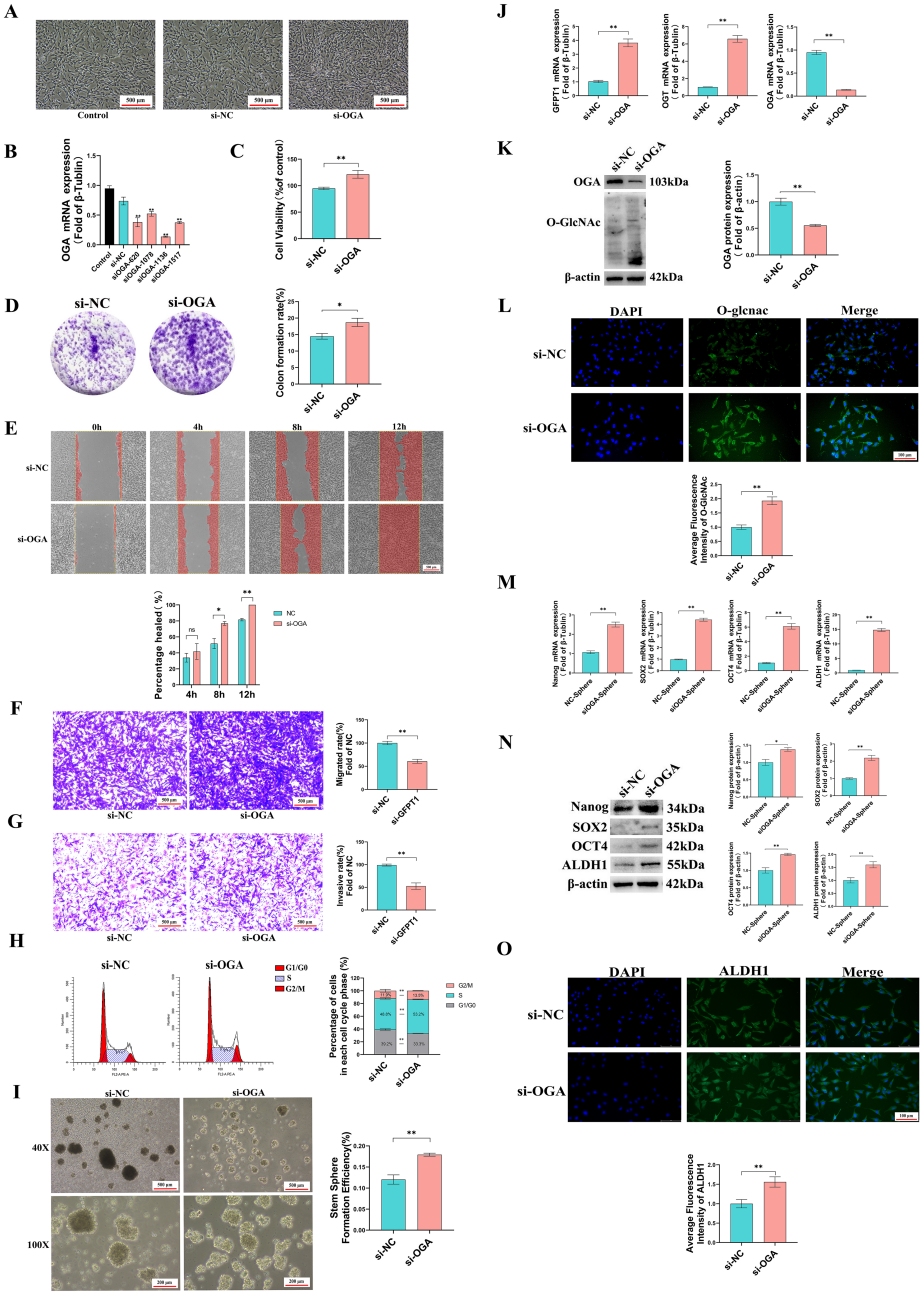
Transient OGA silencing causes accumulation of O-GlcNAc modification and exacerbates high-glucose-induced malignant behaviors and stem-like properties in canine osteosarcoma cells. (**A**) Representative bright-field images of si-NC- and si-OGA-transfected McKinley cells cultured under high-glucose (25.0 mM) conditions; scale bar = 100 μm. (**B**) qRT-PCR analysis of OGA transient silencing efficiency in adherent cells. (**C**) CCK-8 assay measuring relative cell viability between si-NC and si-OGA groups. (**D**) Representative images of crystal violet-stained colonies and quantitative bar graph of colony numbers. (**E**) Representative images of wound healing assay at 0, 4, 8, and 12 h, and quantitative bar graph of wound closure rates at each time point. (**F**,**G**) Representative images of crystal violet-stained Transwell migration (**F**) and invasion (**G**) assays, and quantitative analysis of migrated and invaded cell numbers. (**H**) Flow cytometric analysis of cell cycle distribution, with stacked bar graph showing the percentages of cells in G0/G1, S, and G2/M phases. Red solid stands for G1/G0 phase, blue diagonal stripes represent S phase, and another red solid indicates G2/M phase. (**I**) Representative images of tumor spheres at 40× and 100× magnifications, and quantitative analysis of sphere-forming efficiency; scale bar = 500 μm. (**J**) qRT-PCR analysis of GFPT1 and OGT mRNA expression levels in adherent monolayer cells. (**K**) Western blot analysis of OGA and global O-GlcNAcylation protein levels, with β-actin as the loading control; bar graph shows densitometric quantification of OGA normalized to β-actin. (**L**) Immunofluorescence staining of global O-GlcNAcylation in adherent cells. Blue signals indicate DAPI-stained cell nuclei, green signals represent O-GlcNAc modification immunofluorescence, and merged panels combine both two channels. Bar graph shows quantification of mean O-GlcNAc fluorescence intensity; scale bar = 100 μm. (**M**) qRT-PCR analysis of stemness-associated genes NANOG, SOX2, OCT4, and ALDH1 mRNA expression in tumor spheres from si-NC and si-OGA groups. (**N**) Western blot analysis of stemness protein expression in tumor spheres, with β-actin as the loading control; bar graphs show densitometric quantification of target proteins normalized to β-actin. (**O**) Immunofluorescence staining of ALDH1 in adherent monolayer cells, and quantitative analysis of mean ALDH1 fluorescence intensity. Blue signals represent DAPI nuclear staining, green signals represent ALDH1 immunofluorescence, and Merge panels combine the two channels; scale bar = 100 μm. All data are presented as mean ± SD from three independent biological replicates. All statistical significance markers (* *p* < 0.05, ** *p* < 0.01) in this figure indicate comparisons between the si-OGA interference group and the si-NC-negative control group, ns represents no significant difference.

The aforementioned knockdown of GFPT1 and OGT confirmed that inhibition of the HBP/O-GlcNAc pathway attenuates the malignant biological behaviors of canine osteosarcoma cells. OGA, as the sole glycosidase responsible for removing O-GlcNAc modifications, was targeted for transient silencing to reversely validate the functional role of O-GlcNAcylation in mediating high-glucose-induced malignant and stemness phenotypes in osteosarcoma. A si-NC-negative control group was established, and all cellular functional and molecular assays were uniformly conducted under high-glucose (25.0 mM) conditions to investigate the promoting effects of O-GlcNAc modification accumulation on cellular biological behaviors (Figure 6).

Bright-field morphological observation (Figure 6A) revealed a marked difference in proliferation between the two groups: si-NC cells exhibited moderate density with regular monolayer spreading, whereas si-OGA cells displayed denser growth with extensive overlapping and aggregation of spindle-shaped cells, along with a noticeably higher number of mitotic cells, preliminarily suggesting that OGA silencing-induced O-GlcNAc modification accumulation accelerates osteosarcoma cell proliferation.

The knockdown efficiency of si-OGA was first validated by qRT-PCR (Figure 6B). Compared with the si-NC group, si-OGA-transfected cells exhibited a significant reduction in OGA mRNA expression, confirming successful transient knockdown; among the siOGA sequences tested, siOGA-1136 showed the highest silencing efficiency and was therefore selected for subsequent experiments. Concurrent qRT-PCR analysis (Figure 6J) revealed that OGA silencing significantly upregulated the mRNA expression of both GFPT1 and OGT, the two core upstream rate-limiting enzymes of the HBP, suggesting the formation of a positive feedback loop that further amplifies HBP flux and O-GlcNAc substrate supply.

Western blot analysis further confirmed the transcriptional findings at the protein level (Figure 6K). Following si-OGA transfection, OGA protein abundance was markedly reduced, accompanied by a significant global increase in O-GlcNAcylation levels. Densitometric quantification confirmed statistically significant differences in both OGA protein expression and global O-GlcNAcylation levels between the si-OGA and si-NC groups. Immunofluorescence staining for O-GlcNAc modification was performed on adherent monolayer cells to visually assess changes in modification levels following OGA inhibition (Figure 6L). The si-OGA group displayed widespread and intense green fluorescence (representing O-GlcNAc modification), whereas the si-NC group showed moderate to weak fluorescence intensity. Quantitative analysis of mean fluorescence intensity confirmed a significant increase in O-GlcNAc modification signals upon transient OGA knockdown, which was fully consistent with the Western blot results.

CCK-8 assay was performed to quantitatively assess cell proliferation activity (Figure 6C). At all time points examined, the relative cell viability of the si-OGA group was significantly higher than that of the si-NC group, demonstrating that OGA silencing and subsequent O-GlcNAc modification accumulation substantially enhance the proliferative activity of osteosarcoma cells under high-glucose conditions.

Colony formation assay was conducted to evaluate long-term clonogenic survival potential (Figure 6D). After static continuous culture, the si-NC group formed a moderate number of macroscopic colonies, whereas the si-OGA group produced more numerous colonies with larger individual sizes and more densely packed internal cellular organization. Quantitative analysis confirmed a significant increase in colony-forming efficiency upon OGA silencing compared with the si-NC group.

Wound healing assay was performed to quantify horizontal cell migration by recording the remaining wound area at 0, 4, 8, and 12 h post-scratching (Figure 6E). At all four time points, the wound closure rate of the si-OGA group was consistently higher than that of the si-NC group, with the migratory gap between the two groups progressively widening over time. At 4 h, no obvious difference in wound gap was observed between the two groups; at 8 h, the si-OGA group achieved approximately 50% closure while the si-NC group reached about 40%; at 12 h, the si-OGA wounds were nearly completely closed, whereas the si-NC group achieved approximately 90% closure. Statistical significance at each time point (* *p* < 0.05, ** *p* < 0.01) was determined relative to the si-NC-negative control group.

Transwell migration and invasion assays were performed to assess unhindered vertical migration and basement membrane invasion, respectively (Figure 6F,G). Following 24 h of serum-gradient chemotactic incubation and crystal violet staining, cell counts in 200× fields revealed that the number of migrated and invaded cells in the si-OGA group was markedly increased compared with the si-NC group. Quantitative analysis confirmed statistically significant differences between the two groups, consistent with the wound healing results, collectively demonstrating that OGA silencing-mediated elevation of O-GlcNAc modification simultaneously enhances both horizontal migration and extracellular matrix invasion of osteosarcoma cells.

Flow cytometric analysis with PI staining was performed to examine cell cycle distribution following OGA silencing (Figure 6H). Compared with the si-NC group, the si-OGA group exhibited accelerated G1-to-S phase transition, with significantly increased proportions of S and G2/M phase cells and a corresponding decrease in G0/G1 phase cells. The stacked bar chart visually depicts this cell cycle acceleration, indicating that OGA knockdown relieves proliferative arrest and accelerates cell division.

Tumor sphere formation assay was performed under ultra-low attachment conditions to enrich for stem cell subpopulations in a high-glucose microenvironment (Figure 6I). At 40× and 100× magnifications, the si-OGA group formed abundant large, structurally intact tumor spheres with smooth and compact edges, whereas the si-NC group produced fewer and smaller spheres with loosely attached peripheral cells. Quantitative analysis of sphere-forming efficiency confirmed that transient OGA silencing significantly enhances the self-renewal and enrichment capacity of stem-like tumor cells, with statistically significant differences compared with the si-NC group.

Total RNA and protein were extracted from tumor spheres of both groups to examine the mRNA and protein expression levels of the core stemness transcription factors NANOG, SOX2, OCT4, and the functional stemness marker ALDH1. qRT-PCR results (Figure 6M) demonstrated that the mRNA abundances of all four stemness genes were significantly upregulated in si-OGA spheres compared with si-NC spheres. Western blot analysis (Figure 6N) revealed fully consistent trends: transient OGA silencing markedly increased the relative protein expression of NANOG, SOX2, OCT4, and ALDH1, with statistically significant differences for each marker compared with the si-NC group.

ALDH1 single-label immunofluorescence staining was performed on adherent monolayer cells as a complementary assessment of stemness phenotypes (Figure 6O). The si-OGA group exhibited uniform and intense green ALDH1 fluorescence signals, whereas the si-NC group showed markedly diminished fluorescence intensity. Quantitative analysis of mean fluorescence intensity confirmed that OGA knockdown increases the abundance of ALDH1-positive stem-like cell subpopulations, corroborating the molecular findings from tumor spheres.

Collectively, transient silencing of the de-modifying enzyme OGA blocked O-GlcNAc removal, resulting in global O-GlcNAc modification accumulation and feedback upregulation of upstream GFPT1 and OGT expression, thereby amplifying overall HBP activity. A comprehensive series of functional assays demonstrated that OGA silencing significantly enhances proliferation, long-term clonogenic survival, horizontal migration, matrix invasion, cell cycle progression, and cancer stem cell self-renewal in canine osteosarcoma cells under high-glucose conditions. Concurrently, the classical stemness markers NANOG, SOX2, OCT4, and ALDH1 were substantially upregulated at both transcriptional and protein levels. All statistical comparisons in this figure were performed relative to the si-NC-negative control group. Collectively, these results, together with the aforementioned GFPT1 and OGT knockdown experiments, form a complete bidirectional loss- and gain-of-function validation, collectively demonstrating that elevated O-GlcNAc modification levels are sufficient to drive high-glucose-induced malignant progression and maintenance of stemness properties in osteosarcoma.

### 3.4. In Vivo Validation of O-GlcNAcylation in Regulating Stemness of Canine Osteosarcoma Cells

**Figure 7:**
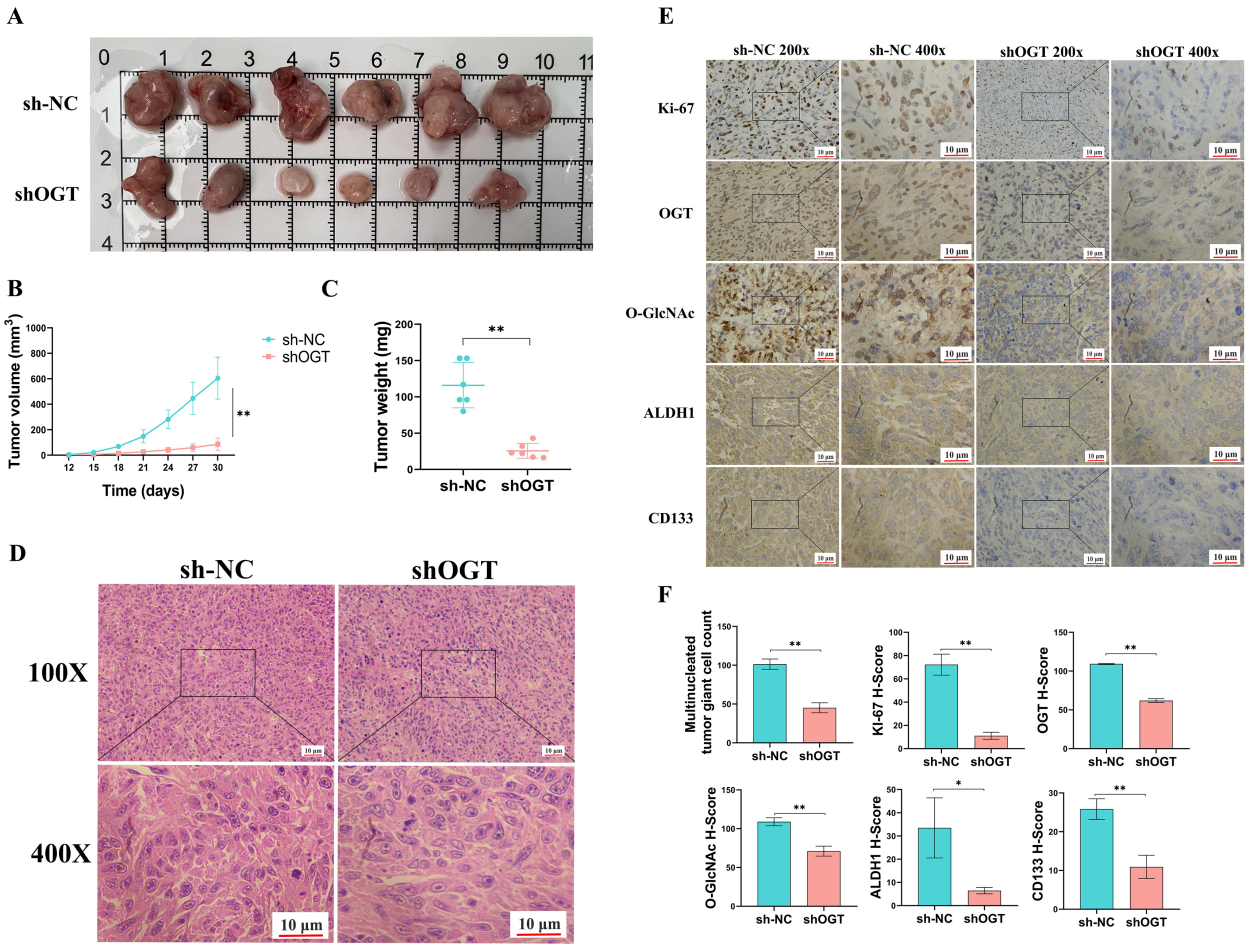
Stable OGT knockdown suppresses tumor growth, malignant pathological features, and stemness marker expression in canine osteosarcoma xenografts in nude mice. (**A**) Gross photographs of subcutaneous tumors excised from nude mice 30 days after inoculation with sh-NC or sh-OGT cells. (**B**) Dynamic tumor volume growth curves of the two groups monitored continuously over 30 days. (**C**) Scatter bar graph showing tumor weights at the experimental endpoint for both groups. (**D**) H&E staining of tumor tissues from both groups, showing cellular morphology at 100× and 400× magnifications; scale bar = 10 μm. (**E**) Representative immunohistochemical staining images of Ki-67, OGT, O-GlcNAc, ALDH1, and CD133 in tumor tissues at 200× and 400× magnifications; scale bar = 10 μm. (**F**) Quantification of multinucleated tumor giant cells and semi-quantitative H-Score analysis of immunohistochemical staining for Ki-67, OGT, O-GlcNAc, ALDH1, and CD133. All data are presented as mean ± SD from three independent biological replicates. All statistical significance markers (* *p* < 0.05, ** *p* < 0.01) in this figure indicate comparisons between the sh-OGT group and the sh-NC-negative control group.

To validate the in vivo regulatory role of OGT-mediated O-GlcNAc modification in osteosarcoma tumorigenesis, malignant proliferation, and stemness properties, sh-NC-negative control and sh-OGT stable knockdown canine osteosarcoma cells were subcutaneously injected into the axillary region of nude mice to establish a xenograft tumor model. Tumor growth was continuously monitored for 30 days, and at the experimental endpoint, tumors were completely excised for gross weighing, histopathological HE staining, and immunohistochemical detection of proliferation, pathway, and stemness markers (Figure 7). Tumor weight and volume growth statistics of nude mice are displayed in Supplementary Table S4.

Gross morphological observation of the excised tumors (Figure 7A) revealed that the sh-NC control group developed larger, firmer, and well-vascularized tumors, whereas the sh-OGT knockdown group exhibited significantly smaller and shriveled tumors, with a visually discernible difference between the two groups.

Dynamic tumor growth curves over the 30-day monitoring period (Figure 7B) showed that tumor volumes began to diverge between the two groups from day 12 post-inoculation, with the difference progressively widening over time. After day 21, the sh-NC group exhibited rapid tumor volume expansion, while the sh-OGT group showed significantly retarded growth. At the 30-day endpoint, tumor volumes in the sh-OGT group were significantly lower than those in the sh-NC group (*p* < 0.01).

Tumor weight analysis at the experimental endpoint (Figure 7C) was fully consistent with the volume changes: the average tumor weight in the sh-NC group was significantly higher than that in the sh-OGT group, with a highly statistically significant difference, demonstrating that stable OGT knockdown significantly suppresses the in vivo proliferative and tumorigenic capacity of osteosarcoma cells. All statistical significance markers in this figure indicate comparisons between the sh-OGT group and the sh-NC group.

HE staining was performed on paraffin-embedded tumor sections (Figure 7D), and histopathological morphology was examined at 100× and 400× magnifications. The sh-NC group exhibited disorganized tumor cell arrangement with pronounced cellular atypia, heterogeneous and hyperchromatic nuclei, abundant pathological mitotic figures, and numerous multinucleated tumor giant cells, displaying typical malignant pathological features of osteosarcoma. In contrast, the sh-OGT group showed markedly reduced atypia, fewer mitotic figures, and significantly decreased multinucleated tumor giant cells, with relatively orderly cell arrangement. Quantitative analysis of multinucleated tumor giant cells (Figure 7F) confirmed that the sh-OGT group contained significantly fewer giant cells than the sh-NC group, suggesting that OGT knockdown alleviates the malignant pathological phenotype of osteosarcoma in vivo.

Immunohistochemical staining was performed to detect the expression and localization of the proliferation marker Ki-67, the pathway core protein OGT, global O-GlcNAc modification, and the stemness markers ALDH1 and CD133 in tumor tissues. Images were captured at 200× and 400× magnifications, and staining intensity and positive cell proportions were semi-quantitatively assessed using the H-Score system (Figure 7E,F). Ki-67 staining revealed abundant brownish-yellow positive signals within nuclei in the sh-NC group, with extremely high H-Scores, whereas the sh-OGT group showed substantially fewer positive cells and significantly reduced scores, indicating that OGT knockdown suppresses in vivo tumor cell proliferation. OGT and O-GlcNAc staining showed strong positive signals in the cytoplasm and nuclei of tumor cells in the sh-NC group, with significantly higher H-Scores compared with the sh-OGT group. The sh-OGT group exhibited markedly attenuated staining intensity and reduced positive cell proportions, confirming that OGT knockdown concurrently reduces global O-GlcNAc modification levels in vivo. Stemness marker staining for ALDH1 and CD133 revealed extensive positive staining in the sh-NC group with significantly elevated H-Scores, whereas the sh-OGT group showed sparse and weaker positive signals with significantly downregulated expression levels. H-Score statistical analysis for all five markers demonstrated that the sh-OGT group scored significantly lower than the sh-NC group, with statistically significant differences between the two groups (* *p* < 0.05, ** *p* < 0.01). Histopathological scoring of tumor xenograft tissues is provided in Supplementary Table S4.

The in vivo xenograft tumor results were fully consistent with the in vitro cellular findings: stable OGT knockdown significantly suppressed tumor growth, reduced tumor volume and weight, alleviated malignant atypia and multinucleated tumor giant cell formation, and downregulated the expression of the proliferation marker Ki-67, pathway proteins OGT and O-GlcNAc, and the stemness markers ALDH1 and CD133. All statistical significance markers in this figure indicate comparisons between the sh-OGT group and the sh-NC-negative control group, collectively providing in vivo evidence that OGT-mediated O-GlcNAc modification is a critical molecular determinant sustaining proliferation, malignant pathological features, and stem-like properties in osteosarcoma.

### 3.5. O-GlcNAc-Modified Proteomics Screens and Identification of Core Target Proteins Mediating Stemness Regulation in Canine Osteosarcoma Cells

**Figure 8:**
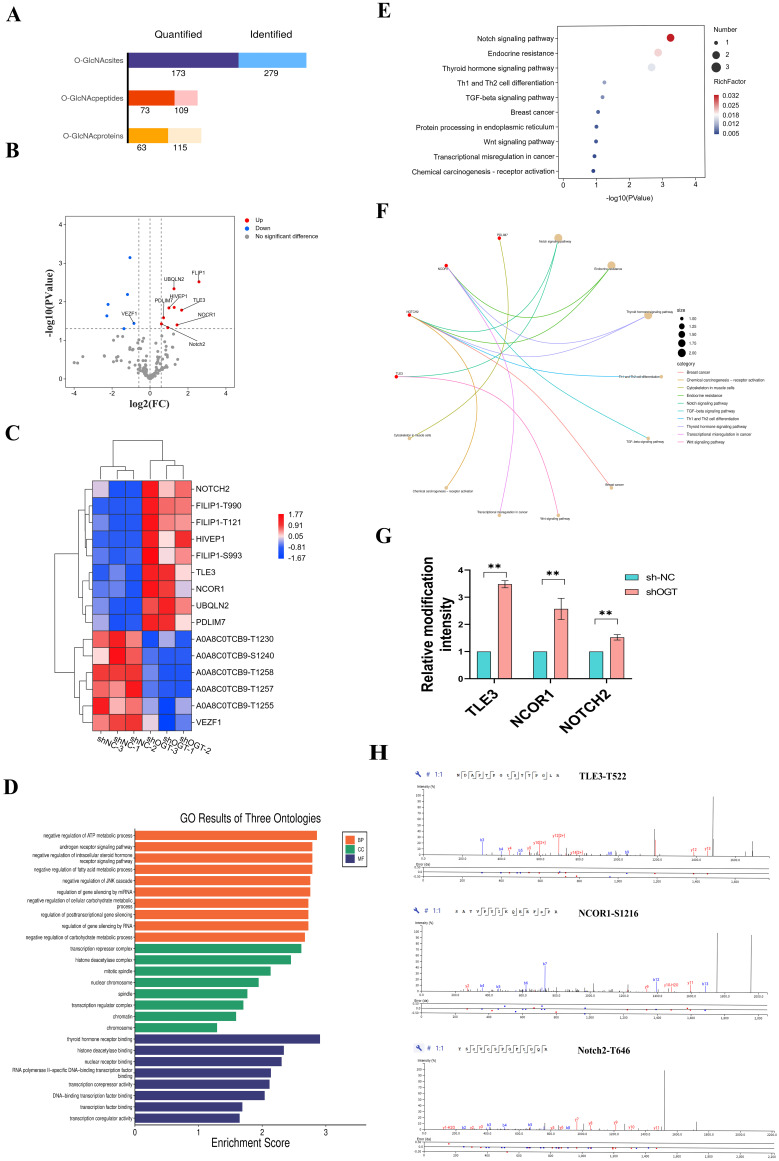
Quantitative O-GlcNAc-modified proteomics analysis of sh-NC vs. sh-OGT cells: identification and validation of core modified target proteins regulating stemness in osteosarcoma. (**A**) Bar chart summarizing the numbers of identified and quantifiable O-GlcNAc modification sites, modified peptides, and modified proteins in this proteomic analysis. (**B**) Volcano plot of differentially O-GlcNAcylated modification sites. Red dots represent significantly downregulated modification sites in the sh-OGT group compared with the sh-NC group, blue dots represent significantly upregulated sites, and gray dots represent sites with no significant difference. Vertical dashed lines at log_2_(FC) = ±1 stand for fold change threshold (|FC| = 2), and the horizontal dashed line at −log_10_(*p*) = 1.3 corresponds to significance threshold *p* = 0.05. (**C**) Hierarchical clustering heatmap of the top 15 key differentially modified proteins. Red indicates high modification abundance, and blue indicates low modification abundance. (**D**) GO functional enrichment bar plot across three ontologies (biological processes, BP; cellular components, CC; molecular functions, MF), with the x-axis representing enrichment scores. Full uncropped high-resolution mass spectrum images are available in Appendix Figure A3. (**E**) KEGG pathway enrichment bubble plot, with the y-axis showing pathway names, the x-axis showing −log_10_(*p*-value), bubble size representing the number of enriched proteins, and color corresponding to enrichment factor. Full uncropped high-resolution mass spectrum images are available in Appendix Figure A4. (**F**) Protein-pathway interaction network, with lines representing interactions between proteins and pathways. Full uncropped high-resolution mass spectrum images are available in Appendix Figure A5. (**G**) Quantitative bar graph of relative O-GlcNAc modification intensity at the key modification sites of TLE3, NCOR1, and NOTCH2. (**H**) Representative raw tandem mass spectrometry spectra of the three modification sites—TLE3-T522, NCOR1-S1216, and NOTCH2-T646—for validation of modification site authenticity. All quantitative data are presented as mean ± SD from three independent biological replicates. Vertical black solid lines mark the characteristic fragment ion peaks of O-GlcNAc modified amino acid residues. Full uncropped high-resolution mass spectrum images are available in Appendix Figure A6. All statistical significance markers in this figure (** *p* < 0.01) indicate comparisons between the sh-OGT group and the sh-NC-negative control group.

To systematically dissect the downstream molecular targets and signaling pathways through which OGT-mediated O-GlcNAc modification regulates stemness properties in canine osteosarcoma cells, sh-NC-negative control and sh-OGT stable knockdown cells were collected under uniform high-glucose culture conditions. Total proteins were extracted and subjected to label-free quantitative O-GlcNAc-modified proteomics analysis. The complete workflow included total protein extraction, tryptic digestion, specific enrichment of O-GlcNAcylated peptides, liquid chromatography fractionation, tandem mass spectrometry data acquisition, modified site identification, quantitative comparison of modification abundance between the two groups, GO and KEGG bioinformatics functional enrichment, and protein-pathway interaction network construction. Additionally, the specific modification sites of the core target proteins identified were validated by raw tandem mass spectrometry spectra, and modification abundance quantification was performed (Figure 8).

The overall statistics of the modified proteomics data (Figure 8A) revealed that a total of 279 O-GlcNAc modification sites, 109 modified peptides, and 115 O-GlcNAcylated proteins were identified in this experiment, among which 173 modification sites, 73 peptides, and 63 proteins exhibited quantitative differences between the two groups. This substantial identification scale provided comprehensive data support for subsequent differential molecule screening and bioinformatics analysis.

A differential volcano plot was constructed based on quantitative data from all modification sites (Figure 8B), with the sh-NC group serving as the reference. The x-axis represents log_2_(fold change) of modification abundance, and the y-axis represents −log_10_(*p*-value). Red dots indicate significantly downregulated modification sites in the sh-OGT group compared with the sh-NC group, blue dots indicate a small number of significantly upregulated sites, and gray dots represent sites without statistically significant differences. The overall distribution is consistent with the biological characteristics of OGT as the sole glycosyltransferase—knockdown of OGT led to a concomitant decrease in O-GlcNAc modification abundance for the vast majority of proteins. Among them, proteins involved in transcriptional regulation and stem cell homeostasis, including TLE3, NCOR1, NOTCH2, FILIP1, HIVEP1, UBQLN2, and VEZF1, exhibited the most prominent downregulation in modification levels and were thus identified as core candidate downstream substrates in this study.

A hierarchical clustering heatmap was generated for the top 15 differentially modified proteins (Figure 8C). Color gradients represent relative modification abundance (red indicating high modification levels and blue indicating low modification levels), with two biological replicate samples per group arranged horizontally and proteins clustered vertically according to modification expression patterns. The heatmap clearly showed that core stemness regulatory proteins—including TLE3, NCOR1, NOTCH2, FILIP1 family members, and HIVEP1—exhibited intense red high-modification signals in the sh-NC group, whereas all of these proteins transitioned to blue low-modification states in the sh-OGT group. Only a few proteins (e.g., AOA8C0TCB9 and VEZF1) showed slight upregulation of modification levels. The clustering analysis clearly distinguished the significant difference in overall O-GlcNAc modification profiles between the two groups, further identifying TLE3, NCOR1, and NOTCH2 as the three core target proteins for subsequent validation.

GO functional enrichment analysis across three major ontologies was performed on all 63 differentially modified proteins (Figure 8D), with orange bars representing biological processes, green bars representing cellular components, and purple bars representing molecular functions. The x-axis indicates enrichment scores, with higher scores reflecting stronger enrichment significance. At the biological process level, numerous terms were concentrated in transcription negative regulation, RNA biosynthesis regulation, stem cell homeostasis maintenance, cell differentiation inhibition, and epithelial–mesenchymal transition regulation—all stemness-related biological programs. Cellular component enrichment revealed that the differentially modified proteins were predominantly localized to the nucleus, chromatin, and nuclear co-repressor complexes. Molecular function analysis showed significant enrichment in DNA-specific binding, transcription factor binding, and chromatin remodeling repression. Collectively, the GO enrichment results suggest that O-GlcNAc modification maintains osteosarcoma stem cell homeostasis primarily through regulating the function of nuclear transcriptional repressor complexes.

KEGG pathway enrichment analysis was presented as a bubble plot (Figure 8E), with the y-axis representing pathway names and the x-axis representing −log_10_(*p*-value). Bubble size indicates the number of differentially modified proteins enriched in each pathway, and color gradients correspond to enrichment factor values. The most significantly enriched pathway was the Notch signaling pathway, followed by thyroid hormone signaling, Th1/Th2 cell differentiation, TGF-β, and Wnt signaling pathways. Notch and Wnt are core stemness pathways regulating self-renewal and differentiation arrest in osteosarcoma stem cells, while TGF-β synergistically mediates invasive phenotypes. The convergence of multiple pathways forms a stemness regulatory network, indicating that O-GlcNAc modification exerts its function through global regulation of core proteins across multiple stemness-associated pathways.

A protein-pathway interaction network was constructed for all differentially modified proteins (Figure 8F), with the number of connecting lines reflecting the confidence of interactions between proteins and pathways. TLE3, NCOR1, and NOTCH2 occupied the central hub positions of the entire network, exhibiting strong direct interactions with each other while radiating outward to associate with numerous transcription-regulatory modified proteins, further supporting their roles as key downstream substrates through which the HBP/O-GlcNAc signaling axis regulates stemness in osteosarcoma.

Quantitative analysis of modification abundance was performed for three specific modification sites—TLE3-T522, NCOR1-S1216, and NOTCH2-T646 (Figure 8G). All statistical comparisons were made relative to the sh-NC group. OGT knockdown led to a significant reduction in O-GlcNAc modification intensity for all three target proteins, quantitatively confirming that OGT deficiency substantially attenuates the glycosylation levels of these three core proteins.

To exclude false-positive interference from mass spectrometry, complete LC-MS/MS raw tandem mass spectra for the three modification sites (TLE3-T522, NCOR1-S1216, and NOTCH2-T646) were presented (Figure 8H). The N-terminal b ions and C-terminal y ions of each target peptide precisely matched theoretical molecular masses, with mass errors for all characteristic fragment ions stably controlled within ±0.5 Da. The characteristic +203 Da mass shift corresponding to HexNAc modification was clearly identifiable in the spectra, providing direct mass spectrometric evidence confirming the authenticity and reliability of the three modification sites.

In summary, through systematic comparison between sh-NC-negative control and sh-OGT stable knockdown cells, this modified proteomics analysis screened and validated three key downstream target proteins—TLE3, NCOR1, and NOTCH2. Bioinformatics enrichment analysis confirmed that the differentially modified proteins were predominantly concentrated in Wnt, Notch, and other stemness-associated signaling pathways. This study elucidates the molecular mechanism from the perspective of post-translational modification: high glucose induces OGT activation, which promotes O-GlcNAc modification of TLE3, NCOR1, and NOTCH2, thereby modulating downstream Notch/Wnt pathways and ultimately maintaining stemness properties in canine osteosarcoma cells. All statistical significance markers in this figure indicate comparisons between the sh-OGT group and the sh-NC-negative control group.

## 4. Discussion

In this study, we systematically investigated the molecular mechanisms by which the high-glucose microenvironment regulates stemness in canine osteosarcoma cells via the HBP/O-GlcNAc signaling axis. By integrating untargeted metabolomics with O-GlcNAc-modified proteomics, we identified the key metabolic nodes and core effector proteins of this pathway. Cellular functional assays demonstrated that high glucose significantly enhanced proliferation, migration, invasion, and tumor sphere-forming capacity, and upregulated the expression of stemness markers including SOX2, NANOG, OCT4, and ALDH1. Metabolomics further confirmed the aberrant activation of the HBP in canine osteosarcoma cells. To verify the direct regulatory effect of the high-glucose microenvironment on the HBP pathway, we measured intracellular UDP-GlcNAc levels and key HBP/O-GlcNAc pathway indicators under different glucose concentrations. The results showed that all these indicators were significantly upregulated with increasing glucose concentrations, indicating that high glucose enhances HBP flux and O-GlcNAcylation in a dose-dependent manner. Building on these findings, we performed loss-of-function experiments by silencing GFPT1 or OGT, and found that inhibition of the HBP/O-GlcNAc pathway significantly reduced O-GlcNAcylation levels and suppressed stemness, whereas OGA silencing enhanced O-GlcNAcylation and promoted stemness. To validate these in vitro findings, we established a subcutaneous xenograft model in nude mice, which confirmed that OGT knockdown-mediated reduction of O-GlcNAcylation significantly inhibited tumor growth, alleviated malignant pathological features, and downregulated intratumoral proliferation and stemness markers. Finally, O-GlcNAc-modified proteomics identified a panel of key target proteins, including TLE3, NOTCH2, and NCOR1, which were predominantly enriched in stemness-associated pathways such as Wnt and Notch signaling, suggesting that they may serve as critical downstream effector molecules through which the HBP/O-GlcNAc axis regulates osteosarcoma stemness. Collectively, this study systematically elucidates a complete signaling cascade of “high glucose → HBP flux ↑ → O-GlcNAcylation ↑ → stemness pathway activation → malignant progression,” revealing a novel mechanism by which high glucose regulates canine osteosarcoma stemness at the interface of metabolism and post-translational modification, and providing a theoretical foundation for understanding malignant progression and exploring targeted therapeutic strategies in canine osteosarcoma.

The high-glucose microenvironment is a critical extrinsic factor driving malignant phenotypes by enhancing proliferation, invasion, and stemness through metabolic reprogramming [24,25]. To more closely approximate canine physiology and tumor microenvironment characteristics, we established glucose gradients of 5.5 mM (simulating fasting hypoglycemia), 11.1 mM (simulating postprandial physiological control), and 25.0 mM (simulating pathological hyperglycemia). Our results demonstrated that, compared with 11.1 mM normal glucose, 25.0 mM high glucose significantly enhanced sphere-forming capacity, clonogenic ability, migration, and invasion, while accelerating cell cycle progression from G1 to S phase. Conversely, 5.5 mM low glucose induced G1 phase arrest and suppressed these malignant phenotypes. Furthermore, high glucose significantly upregulated the expression of the core stemness transcription factors SOX2, NANOG, OCT4, and ALDH1. These findings are consistent with reports on human osteosarcoma, breast cancer, and hepatocellular carcinoma, where high glucose promotes stemness [17,37], suggesting that glucose concentration is a critical upstream signal regulating osteosarcoma stemness, and that this mechanism is conserved between humans and canines. Our results further support the use of canine osteosarcoma as a comparative medicine model for human osteosarcoma.

Untargeted metabolomics revealed that the metabolic profiles of canine osteosarcoma cells were distinctly separated from those of normal osteoblasts, with differentially abundant metabolites predominantly enriched in pathways including central carbon metabolism in cancer, alanine–aspartate–glutamate metabolism, amino sugar and nucleotide sugar metabolism, glycine–serine–threonine metabolism, and purine metabolism, indicating that osteosarcoma development is accompanied by global metabolic reprogramming [38,39]. These pathway aberrations do not occur in isolation but are interconnected, converging on the hexosamine biosynthetic pathway (HBP)—the central hub linking glucose metabolism to protein O-GlcNAcylation. Metabolomics showed that key HBP intermediates, particularly UDP-N-acetylhexosamine, were significantly elevated. At the molecular level, the mRNA and protein expression of the glucose transporter GLUT1, the HBP rate-limiting enzyme GFPT1, and the O-GlcNAc transferase OGT were all significantly higher in canine osteosarcoma cells than in normal osteoblasts, establishing a chain-like activation pattern of “substrate supply → pathway rate-limiting → modification catalysis” [40,41]. Following targeted silencing of GFPT1, global O-GlcNAcylation decreased, and proliferation, colony formation, migration, invasion, and sphere-forming capacity were all significantly suppressed, accompanied by downregulation of the core stemness transcription factors SOX2, NANOG, OCT4, and ALDH1, as well as G1 phase arrest. These findings indicate that HBP activity directly participates in stemness maintenance and malignant progression [42,43]. In combination with the glucose gradient intervention results, the high-glucose microenvironment promotes glucose influx via GLUT1 upregulation, channels glucose into the HBP, increases UDP-GlcNAc production and O-GlcNAcylation, thereby upregulating stemness factor expression and promoting stem cell self-renewal and S phase activation. Blockade of GFPT1 reverses these effects, demonstrating that high glucose utilizes the HBP as a metabolic hub to drive stemness and malignant behaviors in canine osteosarcoma through a cascade model of “high glucose → glucose uptake ↑ → HBP flux ↑ → O-GlcNAcylation ↑ → stemness pathway activation.” This mechanism has also been reported in colon cancer, hepatocellular carcinoma, and non-small cell lung cancer, where high glucose induces aberrant glycosylation via HBP to promote proliferation and invasion, a process reversible by HBP inhibitors such as azaserine [43]. In CD133^+^ stem-like cells of hepatocellular carcinoma, HBP is a significantly differentially regulated metabolic pathway, and its activation maintains the stem cell phenotype [35]. Collectively, these lines of evidence support the HBP as a critical mediator between the high-glucose microenvironment and osteosarcoma stemness evolution, suggesting that targeting GFPT1/OGT may represent an effective metabolic intervention strategy for both canine and human osteosarcoma [44,45,46].

To directly assess the impact of O-GlcNAcylation levels on malignant phenotypes and stemness in canine osteosarcoma cells, we employed a bidirectional intervention strategy: OGT knockdown to reduce modification levels and OGA knockdown to elevate them. Functional results showed that OGT knockdown significantly inhibited proliferation, colony formation, migration, invasion, and sphere-forming capacity, and induced cell cycle arrest at G1 phase. In contrast, OGA knockdown enhanced these malignant phenotypes, accelerated G1/S transition, and promoted sphere formation and self-renewal. Meanwhile, O-GlcNAcylation levels positively regulated the expression and nuclear enrichment of the core stemness transcription factors SOX2, NANOG, OCT4, and ALDH1: high modification promoted their nuclear localization and transcriptional activity, whereas low modification led to cytoplasmic retention and increased degradation. These results confirm that O-GlcNAcylation levels directly determine the stemness strength and cell cycle rhythm of canine osteosarcoma cells, and this mechanism is further supported by clinical osteosarcoma samples, where high OGT expression or low OGA expression is significantly associated with poor prognosis, chemoresistance, and metastatic risk [47,48,49,50,51]. Integrating the finding that HBP activation upregulates O-GlcNAcylation, our study fully elucidates a cascade axis of “high-glucose microenvironment → HBP flux ↑ → O-GlcNAcylation ↑ → nuclear enrichment of stemness factors/cycle reprogramming → enhanced malignant phenotypes,” establishing O-GlcNAcylation as a key molecular switch linking metabolic signals to the osteosarcoma stemness program. Targeting OGT/OGA may therefore represent an effective metabolic intervention strategy for osteosarcoma. Based on the in vitro bidirectional intervention results, we concurrently performed in vivo functional validation using sh-NC and sh-OGT subcutaneous xenograft models in nude mice. The results were highly consistent with the in vitro findings: OGT knockdown-mediated reduction of O-GlcNAcylation significantly inhibited xenograft tumor growth, improved malignant pathological features such as atypical hyperplasia, and downregulated intratumoral expression of the proliferation marker Ki-67 and the stemness markers ALDH1 and CD133, thus corroborating at the animal level that O-GlcNAcylation positively regulates proliferation, stemness, and in vivo tumorigenic capacity in canine osteosarcoma, and completing the in vitro–in vivo closed-loop evidence for this regulatory axis.

To dissect the downstream molecular mechanisms by which the HBP/O-GlcNAc axis regulates stemness in canine osteosarcoma, we performed, for the first time in canine osteosarcoma, quantitative O-GlcNAc-modified proteomics. By comparing the OGT knockdown group with the control group, we identified a total of 279 modified proteins and 173 modification sites, among which 115 modified proteins and 63 sites were quantifiable, and 12 differentially modified proteins were screened, including TLE3, NOTCH2, NCOR1, UBQLN2, and VEZF1. Bioinformatics analysis revealed that the differentially modified proteins were significantly enriched in biological processes such as transcriptional regulation, protein stability regulation, and cell cycle. KEGG pathway enrichment clearly pointed to the Wnt and Notch signaling pathways—two classical stemness pathways—as well as involvement of the TGF-β signaling pathway and transcriptional dysregulation in cancer [50,51,52]. The Wnt and Notch pathways play core roles in self-renewal, differentiation imbalance, and malignant invasion of osteosarcoma stem cells, and extensive crosstalk exists between them. TGF-β can synergize with Wnt/β-catenin and Notch to form an “EMT signaling network” [53,54]. In bone homeostasis and bone tumors, the interplay among Smad/β-catenin/TCF complexes and co-transcription factors such as NICD/RBP-Jκ/LEF further demonstrates the existence of multi-pathway integrated regulation [55,56,57]. These pathway enrichment results suggest that O-GlcNAc modification does not regulate a single molecule in isolation, but rather reshapes the Wnt/Notch stemness signaling network through global modification, synergizing with pathways such as TGF-β to collectively drive the stemness phenotype in canine osteosarcoma.

Among the differentially modified proteins identified, TLE3, NOTCH2, NCOR1, UBQLN2, and VEZF1 all exhibited significant changes in O-GlcNAcylation levels. TLE3, a transcriptional co-repressor of the Groucho/TLE family, negatively regulates the Wnt pathway by binding to the β-catenin/TCF4 complex and exerts tumor-suppressive functions, with its protein stability being precisely regulated by the ubiquitin-proteasome pathway [50,51]. NOTCH2 is a key receptor in the Notch signaling pathway; upon cleavage by ADAM10/17 and γ-secretase, it releases NICD2, which translocates to the nucleus to activate downstream target genes, promoting cell cycle progression and inhibiting apoptosis [52,58]. NCOR1, as a nuclear receptor co-repressor, mediates chromatin remodeling and transcriptional repression through HDAC3 recruitment, and its function is regulated by multiple post-translational modifications [59]. UBQLN2 participates in substrate recognition within the ubiquitin-proteasome system and plays a critical role in protein quality control; its high expression in osteosarcoma correlates with worse metastasis-free survival [60,61]. VEZF1, a zinc finger transcription factor, regulates angiogenesis, proliferation, and stem cell properties, and is directly targeted and inhibited by miR-382-5p in osteosarcoma [62,63]. These proteins are respectively involved in Wnt-negative regulation, Notch activation, transcriptional repression complexes, protein homeostasis, and angiogenesis, collectively suggesting that O-GlcNAcylation may facilitate the regulation of osteosarcoma malignant phenotypes from multiple perspectives.

Beyond mechanistic elucidation, our findings carry substantial translational implications from a comparative oncology perspective. Canine osteosarcoma shares up to 99% genetic homology with human osteosarcoma, and the hexosamine biosynthetic pathway (HBP)/O-GlcNAc metabolic axis is evolutionarily conserved across mammals. Given that aberrant O-GlcNAcylation has been implicated in sustaining stemness in human hepatocellular carcinoma, glioblastoma, and breast cancer, the regulatory mechanism identified in our canine model is likely applicable to human osteosarcoma and other glucose-addicted malignancies. Clinically, the observed upregulation of GFPT1 and OGT, along with the specific O-GlcNAc modification sites on TLE3 (T522), NCOR1 (S1216), and NOTCH2 (T646), may serve as potential predictive biomarkers for chemoresistance and unfavorable prognosis in osteosarcoma patients. Therapeutically, pharmacological targeting of HBP flux—for instance, using GFPT1 inhibitors such as azaserine derivatives—or selective disruption of O-GlcNAc-mediated stabilization of stemness regulators could represent a promising strategy to resensitize cancer stem cells to conventional chemotherapeutics like doxorubicin and cisplatin. Moreover, given the druggable nature of O-GlcNAc cycling, our O-GlcNAc-modified proteomic dataset provides a valuable resource for developing small-molecule OGT inhibitors, which may not only improve clinical outcomes in canine osteosarcoma patients but also furnish a robust preclinical foundation for future human clinical trials. We acknowledge that the findings are primarily based on a single cell line (McKinley); further validation in additional canine OS cell lines is warranted. Nonetheless, we acknowledge that inter-breed and inter-patient tumor heterogeneity, as well as differences in glucose metabolic profiles between canines and humans, warrant further prospective cohort studies to validate the universality of these candidate targets.

## 5. Conclusions

In summary, this study establishes, for the first time, a causative link between the high-glucose microenvironment and stemness acquisition in canine osteosarcoma through the HBP/O-GlcNAc signaling axis. We demonstrate that high glucose enhances HBP flux, elevates UDP-GlcNAc production and global O-GlcNAcylation, and subsequently activates downstream Wnt/Notch stemness pathways. Through bidirectional genetic interventions and in vivo xenograft assays, we confirm that O-GlcNAc modification serves as a critical molecular switch bridging glucose metabolic reprogramming and stemness maintenance. Moreover, O-GlcNAc-modified proteomics identifies and validates TLE3, NCOR1, and NOTCH2 as core downstream effector targets of this signaling cascade. Collectively, our findings position the HBP/O-GlcNAc axis as a central metabolic-epigenetic hub in osteosarcoma pathogenesis and provide a rationale for novel therapeutic strategies in both canine and human osteosarcoma.

## Figures and Tables

**Table 1:** siRNA-GFPT1 and siRNA-OGA design sequences.

mRNA Gene ID	Sense Strand Sequence (5′ → 3′)	Antisense Strand Sequence (3′ → 5′)
siNC	UUCUCCGAACGUGUCACGUTT	ACGUGACACGUUCGGAGAATT
siGFPT1-322	GGAGUACAGAGGAUAUGAAUTT	AUCAAUUCCUCGUACUCTTT
siGFPT1-530	GGAGAACCCAUCUCUGUCATT	UGACAGGAUUGGGUUCUCCTT
siGFPT1-602	GGAUUUAUCACCAACUACATT	UGUAGUGUGUAUAAUUCCTT
siGFPT1-860	GGUGUACGAAGUGAACAUATT	UAUGUUCACUUCGUACACCTT
siOGA-620	GCACGGGAAUAUGAGAUAGTT	CUAAUCUCAUAUUCCCGUGCTT
siOGA-1078	GGAUAACAUUCAUGCUAAUTT	AUUAGCAUGAAUGUUAUCCTT
siOGA-1136	GGAAGAUCCACAGAACUCATT	UGAGUUCUGUGGAUCUUCCTT
siOGA-1517	GCCACAACUGUAGUCACAATT	UUGUGACUACAGUUGUGGCTT

**Table 2:** List of shRNA-OGT designs.

mRNA Gene ID	Sense Strand Sequence (5′ → 3′)
shNC	TTCTCCGAACGTGTCACGT
shOGT–1000	GCCAATATCAAACGAGAACAG
shOGT–1203	GGGAAACACTCTAAAGGAGAT
shOGT–1494	GAAGAATAGGTTGCCATCTGT

**Table 3:** List of mRNA primer designs.

mRNA Gene ID	Sense Strand Sequence (5′ → 3′)	Antisense Strand Sequence (3′ → 5′)
Nanog	CCCAAGCACCCAACTCTAGG	GGAAGGAAGAGGAGAGACGGT
SOX2	CCTGGACTTCTTTTTGGGGG	TTTCTGCAAAGCTCCTACCG
OCT4	GAGAGGCAACCTGGAGAACA	CACTCGGACCACATCCTTCT
ALDH1	TACGGTGGTGGTCAAACCTG	GAGGGAAACCTGCCTCTTGT
GFPT1	AGCAGTTGGCACAAGACGAG	TTTCTCCCTGTGATCCCCAC
OGT	CCTGCCAAAACACTGGTACG	AACTCAGCTAACCCTGTGCT
OGA	TCCTGGGTCAGGCTGATGTA	CGGCCCGGAGCCCTATTTT
β-tubulin	CATCTCACTTTCTGCCCCGT	GACCTCCCAGAACTTGGCAC

**Figure A1:**
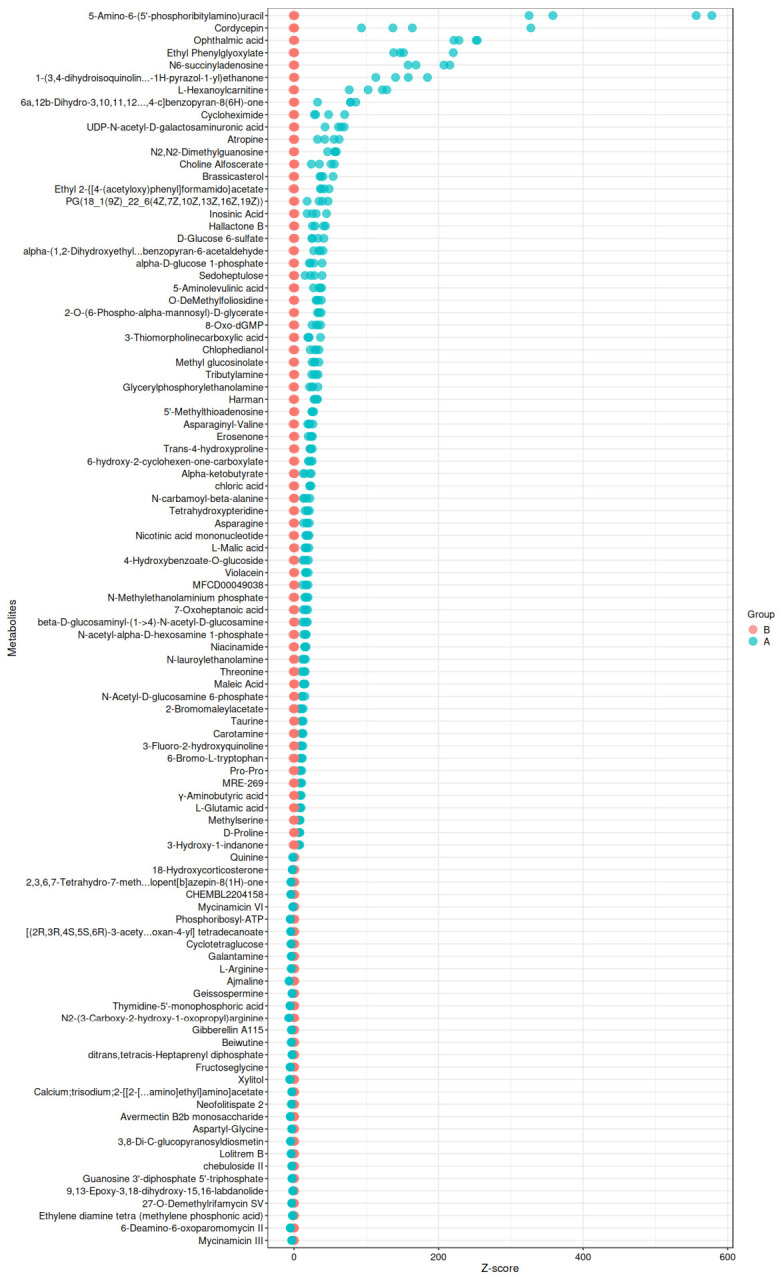
Full-size Z-score distribution plot of differential metabolites (corresponds to Figure 2F). Uncropped high-resolution Z-score scatter plot of all differential metabolites from untargeted metabolomics comparing OBc (Group B, red dots) and OSc (Group A, cyan dots). All metabolite full names are retained in this complete appendix figure. Metabolites abbreviated with ellipses in the plot are listed below.

**Figure A2:**
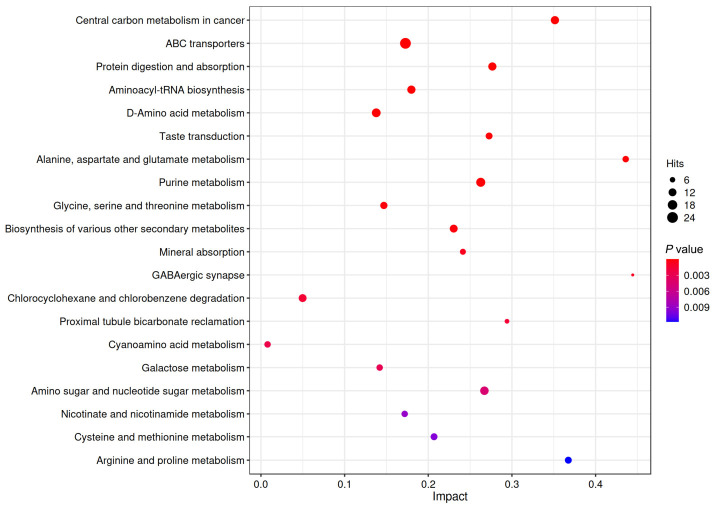
Full-size KEGG enrichment bubble plot of differential metabolites (corresponds to Figure 2G). Complete uncompressed KEGG pathway enrichment bubble diagram for differential metabolites from metabolomic analysis, matching Figure 2G in the main text. X-axis: pathway impact value; dot size = number of enriched differential metabolites; color gradient represents *p*-value significance.

**Figure A3:**
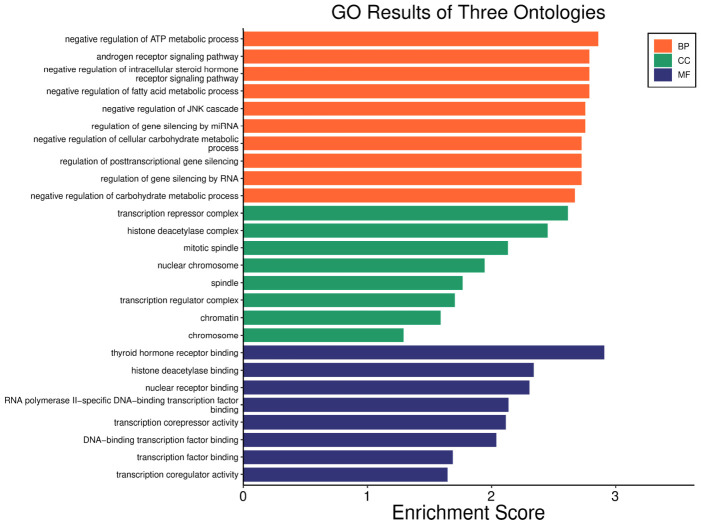
Full GO enrichment bar chart of differential O-GlcNAc modified proteins (corresponds to Figure 8D). Uncropped horizontal bar plot of GO three-ontology enrichment for differentially O-GlcNAc-modified proteins, consistent with Figure 8D in main text. BP (orange), CC (green), MF (purple); X-axis = enrichment score.

**Figure A4:**
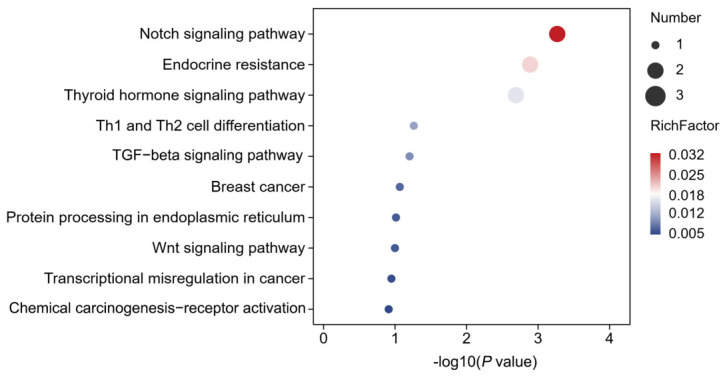
Full KEGG enrichment bubble plot of differential O-GlcNAc modified proteins (corresponds to Figure 8E). Complete KEGG pathway enrichment bubble plot for O-GlcNAc modified proteome (matches Figure 8E). X-axis: −log_10_(*p*-value); dot size = number of enriched modified proteins, color corresponds to RichFactor.

**Figure A5:**
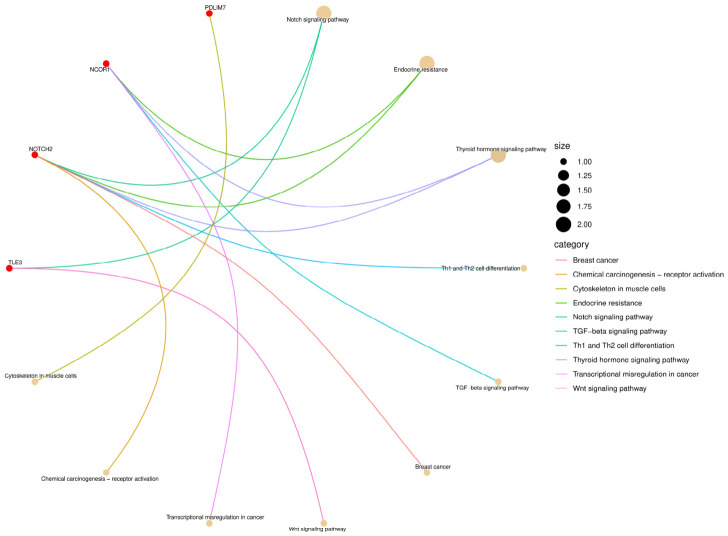
Full interaction network of modified proteins and signaling pathways (corresponds to Figure 8F). Uncompressed protein-pathway correlation network diagram of core O-GlcNAc modified targets and enriched downstream signaling pathways, consistent with Figure 8F in the main text. Node size reflects enrichment weight, colored lines represent regulatory associations.

**Figure A6:**
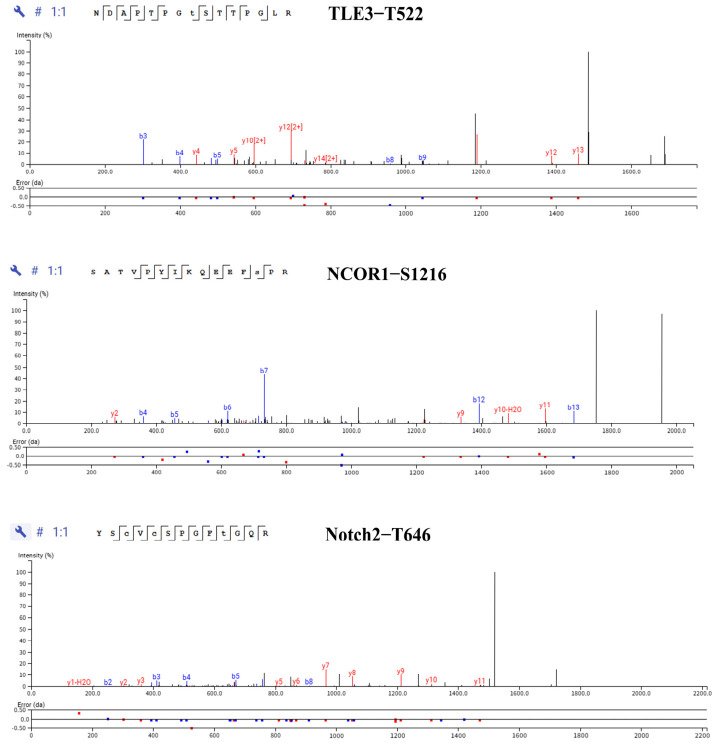
Representative MS/MS spectra of O-GlcNAc modified peptides (corresponds to Figure 8H). Full original mass spectrum graphs of three key O-GlcNAc modification sites (TLE3-T522, NCOR1-S1216, Notch2-T646), which were condensed in Figure 8H of main text. Annotated b-ions (blue) and y-ions (red) are fully displayed for modification site validation. Vertical black solid lines mark the characteristic fragment ion peaks of O-GlcNAc modified amino acid residues.

## Data Availability

The raw data of untargeted metabolomics generated in this study have been deposited in the MetaboLights database and are not publicly available during the peer-review process. Reviewers may access the data via the following dedicated link: https://www.ebi.ac.uk/metabolights/reviewer1a7cd672-570a-42fb-a3c3-264cddaea4f8 (accessed on 6 June 2026). The metabolomics data will be made publicly available upon formal publication of this manuscript. The raw data of O-GlcNAc-modified proteomics have been deposited in the ProteomeXchange database with the dataset identifier PXD079417 and can be accessed via the following link: https://proteomecentral.proteomexchange.org/cgi/GetDataset?ID=PXD079417 (accessed on 8 June 2026).

## Supplementary Materials

The following supporting information can be downloaded at: https://www.mdpi.com/article/10.3390/cells15151359/s1. The complete captions of all Supplementary Tables are listed as follows: Table S1: qPCR quantitative detection data; Table S2: Western Blot protein immunoblot quantification data; Table S3: Immunofluorescence staining statistical results; Table S4: Xenograft tumor formation assay growth statistics; Table S5: Histological analysis of xenograft tumor tissues from nude mice.

## Abbreviations

The following abbreviations are used in this manuscript:

ALDH1	Aldehyde Dehydrogenase 1
APC	Article Processing Charge
BALB/c-nude mice	BALB/c Nude Mice
BCA	Bicinchoninic Acid Assay
BSA	Bovine Serum Albumin
bFGF	Basic Fibroblast Growth Factor
BMI1	B Lymphoma Mo-MLV Insertion Region 1
CCK-8	Cell Counting Kit-8
CD117	Cluster of Differentiation 117
CD133	Cluster of Differentiation 133
cDNA	Complementary Deoxyribonucleic Acid
CHX	Cycloheximide
Control	Blank Control Group
CRediT	Contributor Roles Taxonomy
CSCs	Cancer Stem Cells
Cy3	Cyanine 3
DAB	3,3′-Diaminobenzidine
DAPI	4′,6-Diamidino-2-Phenylindole
DMEM	Dulbecco’s Modified Eagle Medium
ECL	Enhanced Chemiluminescence
EDTA	Ethylene Diamine Tetraacetic Acid
EGF	Epidermal Growth Factor
EMT	Epithelial–Mesenchymal Transition
FBS	Fetal Bovine Serum
FC	Fold Change
FITC	Fluorescein Isothiocyanate
GFPT1	Glutamine-Fructose-6-Phosphate Transaminase 1
GO	Gene Ontology
H&E	Hematoxylin and Eosin Staining
HBP	Hexosamine Biosynthetic Pathway
HG	High Glucose
Hippo-YAP/TAZ	Hippo Signaling Pathway
HRP	Horseradish Peroxidase
HDAC3	Histone Deacetylase 3
IF	Immunofluorescence
IHC	Immunohistochemistry
iProX	China Proteomics Data Repository
KEGG	Kyoto Encyclopedia of Genes and Genomes
LC-MS/MS	Liquid Chromatography-Tandem Mass Spectrometry
LG	Low Glucose
MetaboLights	European Metabolomics Database
NANOG	Nanog Homeobox Protein
NCBI TaxID	NCBI Taxonomy Identifier
NCOR1	Nuclear Receptor Co-Repressor 1
NG	Normal Glucose
NICD2	Notch Intracellular Domain 2
NOTCH2	Notch Receptor 2
O-GlcNAc	O-linked N-acetylglucosamine
OBc	Osteoblast Cells
OGA	O-GlcNAcase
OGT	O-GlcNAc Transferase
OS	Osteosarcoma
OSc	Osteosarcoma Cells
P	P-value
PCA	Principal Component Analysis
PPI	Protein–Protein Interaction Network
PVDF	Polyvinylidene Fluoride Membrane
PXD	ProteomeXchange Dataset Identifier
PMSF	Phenylmethylsulfonyl Fluoride
PBS	Phosphate-Buffered Saline
qPCR	Quantitative Real-Time Polymerase Chain Reaction
RBP-Jκ	Nuclear Binding Protein of Notch Pathway
RIPA	Radio Immunoprecipitation Assay Lysis Buffer
RNA	Ribonucleic Acid
SD	Standard Deviation
sh-NC	short hairpin RNA-Negative Control
sh-OGT	short hairpin RNA targeting OGT
si-GFPT1	small interfering RNA targeting GFPT1
si-NC	small interfering RNA-Negative Control
si-OGA	small interfering RNA targeting OGA
siRNA	small interfering RNA
Smad	Core Molecules of TGF-β Signaling Pathway
SOX2	SRY-Box Transcription Factor 2
SPF	Specific Pathogen-Free
SDS-PAGE	Sodium Dodecyl Sulfate-Polyacrylamide Gel Electrophoresis
TCF4	T-Cell Factor 4
TGF-β	Transforming Growth Factor-β Signaling Pathway
timsTOF Pro	TimsTOF Pro Mass Spectrometer
TLE3	Transducin-Like Enhancer of Split 3
UDP-GlcNAc	Uridine Diphosphate N-Acetylglucosamine
UHPLC-MS/MS	Ultra-High Performance Liquid Chromatography-Tandem Mass Spectrometry
UBQLN2	Ubiquilin-2
VEZF1	Vascular Endothelial Zinc Finger 1
WB	Western Blot
Wnt/β-catenin	Wnt/β-catenin Signaling Pathway
ZEB1	Zinc Finger E-Box Binding Homeobox 1

## Appendix A

The original complete uncropped high-resolution versions of omics-related figures (metabolomics and O-GlcNAc proteomics) are provided in Appendix A for supplementary viewing. Specifically, the full-size raw plots corresponding to Figure 2F,G (untargeted metabolomics of OBc vs. OSc cells), as well as the complete uncompressed enrichment graphs and mass spectrum images supporting Figure 8D–F,H (O-GlcNAc modification proteomics) are placed in this appendix. These full-resolution figures retain all original metabolite names, pathway labels and mass spectrum ion annotations which cannot be fully displayed within the cropped main-text figures due to layout limitations. All appendix figures have been cited at the corresponding positions in the main manuscript.

(1) 1-(3,4-dihydroisoquinolin-1H-pyrazol-1-yl)ethanone (tetrahydroisoquinoline-pyrazole heterocyclic derivative; abbreviated as 1-(3,4-dihydroisoquinolin...-1H-pyrazol-1-yl)ethanone in the figure)
(2) 6a,12b-Dihydro-3,10,11,12-tetrahydroxy-4H-naphtho[1,4-c]benzopyran-8(6H)-one (flavonoid natural product; abbreviated as 6a,12b-Dihydro-3,10,11,12,...4-c]benzopyran-8(6H)-one in the figure)
(3) α-(1,2-Dihydroxyethyl)-3,4-dihydro-2H-benzopyran-6-acetaldehyde (benzopyran derivative; abbreviated as alpha-(1,2-Dihydroxyethyl)...benzopyran-6-acetaldehyde in the figure)
(4) 2,3,6,7-Tetrahydro-7-methoxymethylcyclopenta[b]azepin-8(1H)-one (cyclopenta[b]azepine alkaloid; abbreviated as 2,3,6,7-Tetrahydro-7-meth...lopen[b]azepin-8(1H)-one in the figure)
(5) [(2R,3R,4S,5S,6R)-3-acetyl-2,5-dihydroxy-6-methyloxan-4-yl] tetradecanoate (glycolipid metabolite; abbreviated as [(2R,3R,4S,5S,6R)-3-acety...oxan-4-yl] tetradecanoate in the figure)
(6) Calcium trisodium 2-[[2-(2-hydroxyethyl)aminoethyl]amino]acetate (amino-chelating organic acid metal complex; abbreviated as Calcium;trisodium;2-[[2-[...aminoethyl]amino]acetate in the figure).
